# Comparison of SHANK3 deficiency in animal models: phenotypes, treatment strategies, and translational implications

**DOI:** 10.1186/s11689-021-09397-8

**Published:** 2021-11-16

**Authors:** Jan Philipp Delling, Tobias M. Boeckers

**Affiliations:** 1 Institute for Anatomy and Cell Biology, Ulm University, Albert-Einstein-Allee 11, Ulm, 89081 Germany; 2 Ulm Site, DZNE, Ulm, Germany

**Keywords:** SHANK3, Autism spectrum disorder, ASD, Phelan-McDermid syndrome, PMDS, Therapy

## Abstract

**Background:**

Autism spectrum disorder (ASD) is a neurodevelopmental condition, which is characterized by clinical heterogeneity and high heritability. Core symptoms of ASD include deficits in social communication and interaction, as well as restricted, repetitive patterns of behavior, interests, or activities. Many genes have been identified that are associated with an increased risk for ASD. Proteins encoded by these ASD risk genes are often involved in processes related to fetal brain development, chromatin modification and regulation of gene expression in general, as well as the structural and functional integrity of synapses. Genes of the SH3 and multiple ankyrin repeat domains (*SHANK*) family encode crucial scaffolding proteins (SHANK1-3) of excitatory synapses and other macromolecular complexes. *SHANK* gene mutations are highly associated with ASD and more specifically the Phelan-McDermid syndrome (PMDS), which is caused by heterozygous 22q13.3-deletion resulting in *SHANK3*-haploinsufficiency, or by *SHANK3* missense variants. SHANK3 deficiency and potential treatment options have been extensively studied in animal models, especially in mice, but also in rats and non-human primates. However, few of the proposed therapeutic strategies have translated into clinical practice yet.

**Main text:**

This review summarizes the literature concerning SHANK3-deficient animal models. In particular, the structural, behavioral, and neurological abnormalities are described and compared, providing a broad and comprehensive overview. Additionally, the underlying pathophysiologies and possible treatments that have been investigated in these models are discussed and evaluated with respect to their effect on ASD- or PMDS-associated phenotypes.

**Conclusions:**

Animal models of SHANK3 deficiency generated by various genetic strategies, which determine the composition of the residual SHANK3-isoforms and affected cell types, show phenotypes resembling ASD and PMDS. The phenotypic heterogeneity across multiple models and studies resembles the variation of clinical severity in human ASD and PMDS patients. Multiple therapeutic strategies have been proposed and tested in animal models, which might lead to translational implications for human patients with ASD and/or PMDS. Future studies should explore the effects of new therapeutic approaches that target genetic haploinsufficiency, like CRISPR-mediated activation of promotors.

## Background

Genes of the SH3 and multiple ankyrin repeat domains (*SHANK*) family encode a class of crucial multifunctional scaffolding proteins, whose disruption is highly associated with autism spectrum disorder (ASD) and more specifically the Phelan-McDermid syndrome (PMDS), which results from *SHANK3*-haploinsufficiency or heterozygous *SHANK3* variants that alter function.

### Autism spectrum disorder (ASD)

ASD represents a neurodevelopmental disorder that is highly heritable [1] and heterogeneous, spanning a wide range of clinical manifestations. Generally, ASD is a subgroup within the diagnostic category of “Neurodevelopmental Disorders” in the fifth edition of the American Psychiatric Association’s Diagnostic and Statistical Manual of Mental Disorders (DSM-5). Core symptoms that characterize ASD comprise persistent deficits in social communication and interaction, as well as restricted, repetitive patterns of behavior, interests, or activities. Additionally, sensory anomalies and varying levels of intellectual disability are frequently observed. The symptoms must be present in early childhood and cause clinically significant functional impairment [2]. Furthermore, ASD can occur concurrently with other psychiatric or neurological disorders. Among these comorbidities, anxiety, depression, attention-deficit hyperactivity disorder (ADHD), and epilepsy are diagnosed rather frequently in ASD patients [3]. The severity of symptoms is influenced by environmental as well as genetic factors and covers a wide range of possible manifestations from subtle social deficits, to intellectual disability or severely affected linguistic skills [4, 5].

Commonly ASD is perceived as a rare condition, which is at odds with the estimated prevalence of 1 in 132 in the 2010 Global Burden of Disease study, equating to 52 million cases globally [6]. Gender ratios reported in cohorts of ASD patients range from 2:1 to 5:1 [7, 8] implying a more frequent occurrence in males.

The abovementioned heterogeneity originates from a wide range of genetic and nongenetic etiologies, which are often unknown. The underlying pathophysiological mechanisms that lead to ASD-associated phenotypes are also not fully understood. The fact that twin and family studies consistently demonstrate high concordance rates and a heritability ranging from 40 to 90% provides convincing evidence for a large genetic contribution to ASD [9–11]. In fact, ASD ranks amongst the most heritable medical diagnoses [1]. Fittingly, the number of genes and genomic regions, which are associated with ASD is estimated to be in the hundreds [12–15]. The size of these genetic changes ranges from a single nucleotide to DNA-segments stretching up to millions of bases known as copy number variations (CNV) [12, 13, 16–18]. It has been estimated that 10–20% of ASD patients are affected by rare point mutations or CNVs, most of them de novo [14, 16, 19].

ASD risk genes are often involved in many functional processes that unfold spatiotemporally across development and various brain regions. Despite the obvious challenges of disentangling the underlying dynamics of such multifunctional genes that might also be sex-dependent, converging pathways have been identified. Proteins encoded by ASD risk genes are mostly involved in processes related to fetal brain development, chromatin modification, and regulation of gene expression in general, as well as the structural and functional integrity of synapses. [15, 16, 19–26]. Among others *SHANK1-3* and genes from the *NLGN*- and *NRXN*-family encode synaptic proteins that are crucial for the development or functioning of brain circuits, which contribute to ASD etiology [27–29]. Disruption of ASD-associated genes like *NLGN1* [30], *NLGN2* [31, 32], *NLGN3* [33–37], *NLGN4X* [38, 39], *NRXN1* [40–42], or *NRXN2* [43, 44], but also *CACNA1C* [45], *CNTNAP2* [46], and *GABRB3* [47], is often exploited to replicate ASD phenotypes in animal models. Additionally, several studies have identified glutamatergic neurons during cortical development [48, 49] and the striatum [50] as points of convergence for ASD.

Apart from genetic etiology, environmental factors have been associated with an increased risk of ASD, including hypoxic birth trauma, advanced parental age, maternal obesity, gestational diabetes mellitus, zinc deficiency, and valproate intake during pregnancy [51–54].

### SHANK gene family

**Fig. 1:**
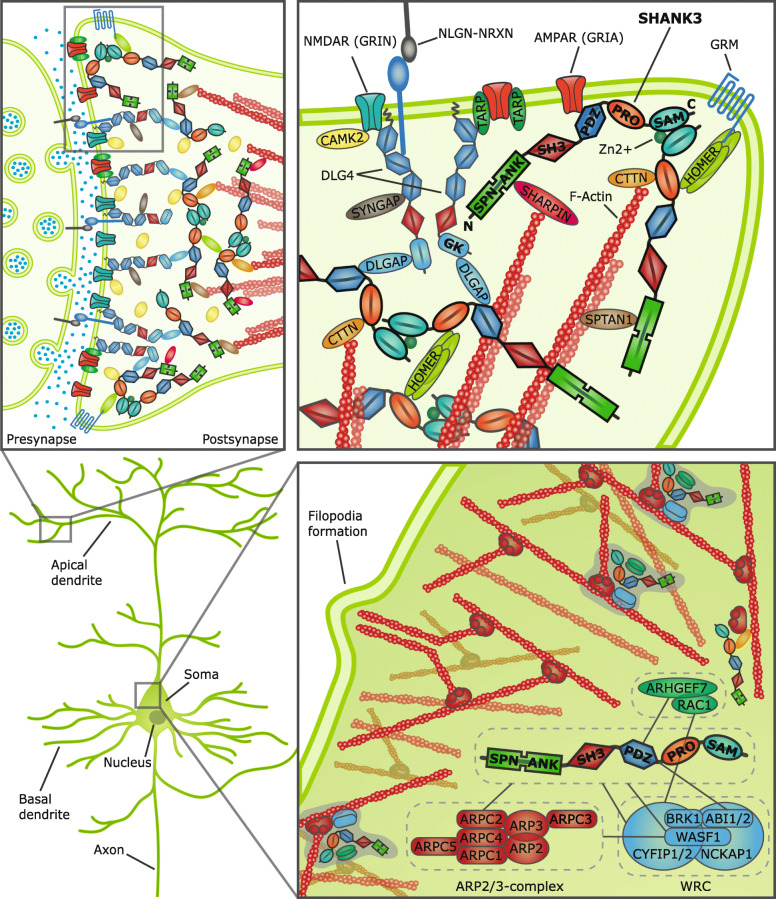
Macromolecular complexes within the interactome of SHANK3. The multiple layers of the functional units in the central nervous system are shown in succession. In the left lower corner a hippocampal pyramidal neuron is depicted. At the top an overview of a synaptic contact and a detailed illustration of the multiple connections between the different interaction domains of SHANK3 and other synaptic proteins is shown. On the right, putative macromolecular assemblies associated to cellular actin nucleation, which include SHANK3, are shown in the soma. SHANK3 localizes to the postsynaptic density (PSD). The PSD is an electron dense thickening underneath the postsynaptic membrane of glutamatergic synapses. It comprises a great multitude of proteins, which form a wide macromolecular complex. Interaction between SHANK3 and other proteins is mediated by six protein domains, which are depicted by form and color in this image: The SPN-, ANK-, SH3-, PDZ-, PRO-, and SAM-domain. A subset of SHANK3-interacting proteins is illustrated here. DLG4 (or PSD95) is also depicted by its functional domains (PDZ, SH3, GK) to emphasize that different proteins can contribute similar domains to the structural composition of the PSD. Membrane-associated guanylate kinases (MAGUKs) such as DLG4 also serve as major organizers of synapses, by forming a modular interface between the multiple layers of the PSD. For instance, DLG4, DLGAP, SHANK3 and HOMER connect GRM and NMDAR complexes. An additional connection of this complex to the cytoskeleton is mediated by SHANK3’s interaction with CTTN, SHARPIN, SPTAN1, and other proteins. Depending on its configuration, the SPN-domain prevents the binding of SPTAN1 to the ANK-domain. The ARP2/3-complex is crucial for actin nucleation and polymerization. By its interaction with the WAVE regulatory complex (WRC), components of the RAC1-pathway, and ARPC2 itself, SHANK3 might mediate their influence on actin dynamics. PSD: Postsynaptic density, SHANK: SH3 and multiple ankyrin repeat domains, SPN: SHANK/ProSAP/N-terminal, SH3: Src homology 3, PDZ: PSD95/DlgA/Zo-1, PRO: Proline-rich, SAM: Sterile alpha motif, ANK: Ankyrin repeats

SHANKs (SHANK1-3), which are also known as proline-rich synapse-associated proteins (ProSAPs), were initially described as proteins that primarily localize to the postsynaptic density (PSD) of excitatory synapses [55–59], which is an electron dense thickening underneath the postsynaptic membrane of glutamatergic synapses. It comprises a great multitude of proteins, which form a wide macromolecular complex that is illustrated in Fig. 1. Among these proteins are other scaffolding and adaptor proteins (e.g., DLGAP1, SHANKs), receptors, channels, and signaling molecules (e.g., NMDAR, AMPAR, GRM, CAMK2), but also cell adhesion proteins like NLGNs or constituents of the cytoskeleton (e.g., actin) [60, 61].

As so-called master scaffolding proteins, which are expressed in the central nervous system (CNS) and peripheral nervous system (PNS, somatic and autonomic), SHANKs interact with and thus arrange intermediate scaffolding proteins at the PSD, which profoundly influences synaptic development and function [55–68]. A quaternary complex in the PSD built from DLG4 (also known as PSD95), DLGAP1 (also known as SAPAP1 or GKAP), SHANK, and HOMER proteins connects metabotropic and ionotropic NMDA type glutamate receptor complexes, which might facilitate their interaction [55–57, 59, 64, 65, 67]. Notably, a rather stable pool of SHANK proteins lies closer to the postsynaptic membrane, while another dynamic pool resides more proximally. The stable but not the dynamic pool seems to be able to bind to DLGAP1 [69].

Through dynamic changes of their molecular composition and chemical modification of synaptic proteins, neuronal synapses are regulated during development and throughout life, altering their shape, quantity, and overall strength [63, 70]. SHANK proteins are crucial in many of these and associated processes. Thus, it is not surprising that mutations leading to dysfunctional synaptic proteins, including SHANKs, result in synaptic and circuitry defects [71]. Accordingly, *SHANK* gene mutations are generally associated with human neuropsychiatric and neurodevelopmental disorders. Defects in *SHANK* genes, but especially *SHANK3*, can be causative for idiopathic ASD and ASD-associated syndromes such as the PMDS, but also schizophrenia and intellectual disability [27, 28, 72–77].

### SHANK3

The *SHANK3* (human) or *Shank3* (rodents) gene is located on chromosome 15E3 in mice, on 7q34 in rats, and on 22q13.3 in humans. *Shank3* is subject to alternative usage of its 6 promotors and additional mRNA splicing [78, 79], resulting in multiple mRNA transcripts and enabling the generation of a great variety of protein isoforms. These findings have led to the prediction of isoform transcripts named *Shank3*a-f, of which *Shank3*a and e are enriched in the striatum, *Shank3*c and d are predominantly expressed in the cerebellum, and *Shank3*b is evenly expressed at very low levels across the brain [79]. Accordingly, western blot analysis has shown an array of different proteins detected by SHANK3 antibodies also depending on their epitopes [80]. However, studies on SHANK3 isoforms have largely focused on mRNA transcripts. Thus, it remains to be clarified how exactly the various transcripts translate into proteins. Nevertheless, this multitude of isoforms enables differential expression patterns across the stages of brain development, brain regions, cell types, and even subcellular structures [79, 81–83], suggesting isoform-specific functions. For example, SHANK3b, which lacks the PRO- and SAM-domain, exhibits nuclear localization, whereas SHANK3a, SHANK3c, and SHANK3e that contain those domains form cytoplasmic clusters [79]. SHANK3a and SHANK3c seem to be the isoforms that primarily localize to excitatory synapses [79]. With recent advances in the prediction of protein structures in silico, it might be possible to determine how the predicted isoforms of SHANK3 differ on a three-dimensional level [84–86].

Tissue-specific expression of these different isoforms is also regulated by epigenetic mechanisms [81, 87, 88]. Notably, DNA methylation within the *SHANK3* gene and isoform expression was altered in human brain tissue of ASD patients [89].

*Shank3* mRNA expression is high in the heart and moderate in the brain and spleen [66]. In the nervous system, *Shank3* mRNA is enriched in the cortex (especially layers 2–4), hippocampus, amygdala, cerebellum (granule cells), striatum, thalamus, spinal cord, and dorsal root ganglia [82, 90–92]. As opposed to the other SHANKs, SHANK3 is highly enriched at cortico-striatal glutamatergic synapses [90].

The interactome of SHANK3 covers a wide variety of proteins, which are involved in many cellular processes [93, 94]. Aside from interactors, which represent a common core interactome involved in scaffolding, processes of the PSD in general, or regulation of the actin cytoskeleton, the majority of interacting proteins varies depending on the brain region. This might enable the functional diversity of SHANK3. Common interactors include the HOMER and DLGAP family, but also actin-associated proteins. Interactors, which are related to the cytoskeleton include subunits of the ARP2/3-complex as major mediator of actin nucleation and constituents of the associated WAVE regulatory complex, such as ABI1, WASF1, and CYFIP2 [93–96]. Interestingly, these common interaction profiles also seem to encompass proteins related to myelin- and mitochondrion-associated processes [96]. Other exemplary categories, which fit the brain-region-dependent SHANK3 interactome, include GTP binding, gluconeogenesis, cell-cell adhesion, or endocytosis [93, 96]. Apart from unbiased proteomic approaches to characterize the interactome of SHANK3, single proteins have been identified, which directly bind to SHANK3. Among these are proteins, which are crucial for dendritic spine formation, synaptic transmission and plasticity, cytoskeleton regulation, and the localization of SHANK3 to the PSD [56, 58, 59, 65, 94, 97–103].

The abovementioned highly complex protein-protein interactions of SHANK3 are mediated by its domains. The longest isoform of SHANK3 in mice comprises six highly conserved domains: SHANK/ProSAP/N-terminal (SPN), ankyrin repeats (ANK), Src homology 3 (SH3), PSD95/DlgA/Zo-1 (PDZ), proline-rich (PRO), and sterile alpha motif (SAM). For instance, the ANK-domain binds to SHARPIN [98], SPTAN1 (also known as *α*-fodrin) [99], and CTNND2 [104], while the PDZ-domain interacts with DLGAP1 (which binds to DLG4) [59], GRIA1 (also known as GluA1 or GluR1 subunit of ionotropic AMPA type glutamate receptors) [100], and CTNNB1 as crucial constituent of the Wnt signaling pathway [105]. A SPN-domain at the N-terminus binds to the ANK-domain and limits its ability to interact with SHARPIN or SPTAN1 [97]. The PRO-region encompasses the binding motifs for HOMER [65, 101] and CTTN [59]. SHANK3’s ability to self-multimerize in a zinc-dependent manner depends on the SAM-domain [59, 102]. Synaptic targeting of SHANK3 is mediated by a conserved C-terminal region that includes the SAM-domain [58], while several nuclear localization signals are responsible for its translocation to the nucleus [105].

Among the remaining interaction partners of SHANK3 are kinases like MAPK1 (also known as ERK2), PRKACA, GSK3B, CSNK2, or RPS6KA2/3 [106, 107]. Notably, murine SHANK3 is phosphorylated by MAPK1 at 18 residues, 3 of which have also been observed in vivo and shown to increase SHANK3 turnover and degradation if phosphorylated. Accordingly, activation of constituents belonging to the MAPK/ERK-pathway, like IGF1R, KIT, PKA, RAF1, MAP2K1, or MAPK1, lead to destabilization of SHANK3. Other proteins like TRIO, TAF1, and SIK1 were shown to stabilize SHANK3 [106]. In addition phosphorylation of a serine at position 685 by PKA facilitates the interaction of SHANK3 with ABI1 [94], while interaction with CTTN is prevented by RPS6KA3-mediated phosphorylation [107].

Generally, it has been shown that SHANK-proteins undergo degradation upon activity-dependent ubiquitination [108], which is influenced by DLGAP1 [109] and regulates their abundance at the synapse.

Aside from its role in postsynapses, SHANK3 also localizes to presynaptic specializations in hippocampal neurons [110], afferent nerve terminals in the spinal cord and peripheral terminals of the skin [92], the neuromuscular junction and the Z-disc of skeletal muscle tissue, where it binds to ACTN2 [111]. Additionally, SHANK3 translocates from synapses to the nucleus in an activity-dependent manner [112] and mediates Ca-dependent signaling to the nucleus via interaction with CAMK2A and L-type calcium channels [113]. Recently, it has also been observed that SHANK3 is involved in TRPV1-mediated pain processing in the dorsal root ganglia and spinal cord [92].

### SHANK-associated ASD and Phelan McDermid syndrome (PMDS)

A connection between *SHANK*-mutations and ASD has been established in several human genetic studies [27, 28, 72–74, 76, 77] and was further validated in animal models targeting *Shank1* [114–117], *Shank2* [118–121], or *Shank3* [78, 80, 82, 83, 90, 92, 94, 118, 122–140]. Behavioral phenotypes, like increased repetitive routines, abnormal social behavior, elevated anxiety levels, impaired neuronal physiology, and altered PSD levels of HOMER, DLGAPs, NMDARs, AMPARs and other proteins, typify SHANK3-deficient murine animal models [78, 83, 90, 94, 118, 122, 124–128, 130, 132]. These resemble some neuropsychiatric disorders in humans. It was estimated in a meta-analysis that approximately 1% of all ASD-cases are accounted for by truncating mutations in the *SHANK* gene family [73]. This is a surprisingly high percentage, considering the etiological diversity of ASD. Notably, no truncating *SHANK1/2/3*-mutations, but mutations, which were predicted to be damaging (PolyPhen-2) were found in 4.7% of the healthy controls [73]. Additionally, *SHANK3* mutations have been observed in both asymptomatic parents and their ASD-diagnosed children [28]. Thus, such non-truncating *SHANK* mutations might not be causative for ASD by themselves, but rather contribute to its development in a susceptible genetic and environmental setting.

The PMDS was the first heterozygous neurodevelopmental disorder associated with *SHANK* mutation [27]. Genetically PMDS is caused by a 22q13.3 deletion and clinically presents with hypotonia, impaired language skills, ASD, and various other symptoms [27, 141, 142]. Apart from classical deletions, the PMDS can be caused by ring chromosomes and unbalanced translocations, but also by *SHANK3* point mutations [73, 143–145]. In nearly all PMDS patients, *SHANK3* is affected, and it is assumed that *SHANK3* haploinsufficiency is the major causative factor of their neurodevelopmental and behavioral deficits, although deletions that do not include *SHANK3* also result in certain subphenotypes of PMDS [142, 146–149]. Genetic screening of patients with ASD, which was not due to a heterozygous loss of the gene, also found *SHANK3* mutations [28, 73, 74, 76, 150]. Many *SHANK3* mutations in humans affect exon 21 and are associated with intellectual disability [73]. Although *SHANK1* and *SHANK2* mutations are also associated with ASD [73, 77], cognitive deficits are more severe in patients with *SHANK3* mutations [73]. Clinical screening for *SHANK* mutations might thus be reasonable, since they represent a potential monogenic and syndromic etiology of ASD [73].

## Main text

### Phenotypes and pathophysiology in animal models of SHANK3 deficiency

Various strategies targeting *Shank3* to mimick pathologies that are observed in PMDS patients have been applied in rodent and non-human primate models. These include the constitutive knockout (KO) models of *Shank3* that affect different exons and thus also a different number its six promotors. These KO strategies result in isoform-specific deletion patterns, leaving some isoforms intact, which might even lead to their compensatory overexpression [125, 151]. Conditional KO-models (cKO) have been used to study the effects of promotor-driven cell-type-specific SHANK3 deficiency, which has connected certain neuron populations or brain regions to subphenotypes of mutant animals. Alternatively, mutations that have previously been associated to ASD in human patients are studied using knock in (KI) strategies. A conditional KI (cKI) model has also been used to reexpress SHANK3 at later stages of development. In this review, all models, which are covered, were assigned a code. The nomenclature used for this code was defined according to the abovementioned strategies of genetic intervention and consists of two parts, which are separated by a vertical bar. The first part of the nomenclature depicts the targeted exons of *Shank3*, and the second represents the affected domain and an associated mutation or a promotor-driven Cre-expression, if present.

An alternative nomenclature would additionally refer to the remaining SHANK3 protein isoforms, since whole exon deletions or point mutations affecting similar regions were reported to result in markedly different isoform compositions. For example, in ex21|PRO mice, the absence of major high-molecular isoforms, increased low-molecular isoforms, and the appearance of a new low-molecular band were observed [125]. Models mimicking point mutations within the same exon presented with different isoform patterns. Mice of the model ex21|PRO-InsG3680 showed an almost complete loss of SHANK3 [127], whereas ex21|PRO-InsG3728 introducing the same mutation described in human patients [28] and a Neo-stop cassette resulted in the loss of major high-molecular isoforms, increased low-molecular isoforms, and the appearance of a new low-molecular band [126], comparable to ex21|PRO-mice. ex21|PRO-R1117X resulted in the loss of major isoforms and the expression of a predicted truncated high-molecular protein [127]. Notably, some missense point mutations, as established in the model ex17|PRM-S685I, do not alter the isoform pattern [94]. An isoform-based nomenclature would be in need of a standardized report on isoform composition across all models investigated, also using antibodies targeting different regions of the protein to cover all of its putative isoforms. This information is currently not available for all models covered in this review. Although it is not feasible to generate a detailed and consistent nomenclature based on protein-isoforms of SHANK3, the models which were summarized under the same term in the nomenclature described above, generally present with similar isoform patterns.

It is important to note that many behavioral studies aim to correlate aberrant behavioral patterns in mutant mice or other model organisms with symptoms in human patients. The interpretation of such behaviors is at least to some extent subjective and sometimes even questionable. Nevertheless, behavioral abnormalities in the animal models covered in this review are categorized according to symptoms and comorbidities, which are frequently observed in ASD or the PMDS. This serves to establish a common ground, which enables comparability between the different animal models and human behavior. Due to the abovementioned subjectiveness, the reader is encouraged to treat those interpretations with caution as one could also argue that rodent behavior should not be humanized.

#### Behavioral traits

##### Social behavior

Among other symptoms, ASD is defined by an impairment of social interacion, which often manifests as difficulties in the approach to social situations, reciprocal social interaction, and verbal but also nonverbal communication. Although PMDS patients often meet the criteria of ASD and display severe impairments of language and communication [152], neural responses to communicative vocal sounds and orienting to social stimuli were less affected in PMDS patients when compared to patients with idiopathic ASD [153]. These differential findings and the fact that an ASD diagnosis is not present in all PMDS patients suggest that SHANK3-deficient animals should not necessarily present with profound social behavioral deficits. Accordingly, such deficits have been reported, however with variability, in SHANK3-deficient animals. Rodent social behavior is highly influenced by experimental conditions and handling. Slight differences in protocols of the most commonly used test, the three-chambered social approach test could also be explanatory for the differences observed between cohorts of animals with identical or similar alterations of the *Shank3* gene.

Here, social behavior was categorized in three subdomains: social motivation/interaction, social recognition, and social communication. For instance, the frequently measured social preference in three-chamber tests or free interaction in social dyads were included in social motivation/interaction, while social novelty preference tasks were categorized as tests of social recognition. Analysis of socially induced ultrasonic vocalizations or social olfactory preference tasks in rodents were regarded as tests of social communication.

Abnormal behavioral patterns concerning social motivation or interaction have been consistently observed in the murine models ex4-22|ALL [128, 130], ex4-9|ANK [78, 80, 124, 154–156], ex11|SH3 [123, 157], ex13-16|PDZ [83, 90, 155, 158–166], and ex14-16|PDZ [131], although deficits were not recapitulated for some of these models in single studies [129, 167, 168]. Other modeling strategies mimicking mutations affecting the PRO-domain or a proline-rich motif (PRM) associated to ABI1, which have been found in ASD-patients, such as ex21|PRO-InsG3680 [127] or ex17|PRM-S685I [94], and ex21|PRO-R1117X [127], harboring a schizophrenia-associated mutation, also resulted in abnormal social interaction behavior. Notably, conditional KO in ex13-16|PDZ-Advillin^Cre^ targeting somatosensory neurons or ex13-16|PDZ-Cdx2^Cre^ targeting the caudal part of the embryo and neural tube, thus sparing the brain, was sufficient to induce aberrant social motivation and interaction [140]. Similarly, SHANK3-deletion in dorsal telencephalic excitatory neurons and glia (ex14-16|PDZ-Emx1^Cre^) [133] or all GABAergic neurons (ex14-16|PDZ-Viaat^Cre^) [131] resulted in such deficits. Cre-expression, and thus deletion of *Shank3* in neocortical excitatory neurons (ex4-22|ALL-NEX^Cre^), *Dlx5/6*-positive GABAergic forebrain neurons (ex4-22|ALL-Dlx5/6^Cre^), which include various subclasses of neocortical interneurons but also MSNs as principal striatal projection neurons [169–174], DRD1- (ex4-22|ALL-Drd1^Cre^) and DRD2-positive neurons (ex4-22|ALL-Drd2^Cre^) [129], did not induce the phenotype previously observed in the constitutive KO model [128], though it has to be noted that such deficits of constitutive KO mice were also not replicated in this study. Mixed evidence and even conflicting results concerning social interaction deficits were observed in the models ex9|ANK [82, 175], ex13|PDZ [139, 176], ex21|PRO [125, 177–179], and the rat model ex6|ANK [134, 180]. Intact social motivation and interaction was described in the analysis of the models ex4-7|ANK [90], ex8|ANK-Q321R [132], ex21|PRO-InsG3728 [126], and ex11-21|SH3-PRO in rats [135].

Another dimension of social behavior is the recognition of previously encountered conspecifics, which can also be deficient if basal social interaction behavior seems to be unaffected.

Such deficits of social recognition were observed in the animals of ex4-22|ALL [128], ex4-7|ANK [90], ex11|SH3 [123], ex13|PDZ [139, 176], ex21|PRO [125, 178, 179], and the rat models ex6|ANK [134] or ex11-21|SH3-PRO [135]. A single study on the model ex21|PRO did not replicate the abovementioned deficits [177]. ASD- or schizophrenia-associated mutations in the murine models ex21|PRO-InsG3680 and ex21|PRO-R1117X [127] elicited deficitary social recognition as well. In conditional KO-models targeting somatosensory neurons in ex13-16|PDZ-Advillin^Cre^ or the caudal embryo, including the neural tube in ex13-16|PDZ-Cdx2^Cre^ abnormal social recognition was recapitulated [140]. Mixed evidence concerning the ability to recognize familiar conspecifics was observed in the model ex13-16|PDZ [90, 155, 161, 164, 166, 168], where some studies found aberrant social recognition, while it seemed to be intact in others. No deficits of social recognition were found in the murine models ex4-9|ANK [78, 80, 167], ex9|ANK [82, 175], ex14-16|PDZ [131], and ex21|PRO-InsG3728 [126]. Similarly, specific deletion of SHANK3 in dorsal telencephalic excitatory neurons and glia (ex14-16|PDZ-Emx1^Cre^) [133], or GABAergic neurons (ex14-16|PDZ-Viaat^Cre^) [131] did not cause dysfunctional social recognition.

The communication of rodents in social contexts is a complex and multidimensional behavior, which is most frequently studied via recordings of ultrasonic vocalizations during social interaction, but can also be analyzed using other modalities.

Abnormalities concerning social communication behaviors were consistently observed in mice of the models ex4-22|ALL [128, 129], ex4-9|ANK [78, 80, 124, 155], ex13-16|PDZ [155, 158, 159, 163], ex14-16|PDZ [131], and ex17|PRM-S685I [94], although single studies did not observe differences compared to wildtype animals [130, 162]. Findings were not consistent in two studies focusing on the rat model ex6|ANK [134, 180]. The models ex9|ANK [82], ex21|PRO [125], and ex11-21|SH3-PRO in rats [135] showed social communication skills, which were undistinguishable from wildtype animals. Mice from the models ex8|ANK-Q321R [132], ex21|PRO-InsG3680 [127], and ex21|PRO-R1117X [127] harboring mutations associated to neuropsychiatric disorder also showed normal social communication in the modalities, which were investigated. Conditional KO in the model ex14-16|PDZ-Viaat^Cre^ targeting all GABAergic neurons [131] replicated these deficits, whereas SHANK3 deletion in dorsal telencephalic excitatory neurons and glia, neocortical excitatory neurons, GABAergic forebrain neurons, or DRD1-/DRD2-positive neurons as established in the models ex14-16|PDZ-Emx1^Cre^ [133], ex4-22|ALL-NEX^Cre^, -Dlx5/6^Cre^, -Drd1^Cre^, or -Drd2^Cre^ [129] did not cause aberrant social communication.

##### Stereotypies

The second set of core symptoms, by which ASD is defined, includes repetitive behaviors with restricted interests and perseveration [2], which are often summarized under the term stereotypy. Such stereotypies can be regarded as the most robust phenotype in many models of SHANK3 deficiency. Excessive grooming with or without development of skin lesions is a commonly observed repetitive behavior in SHANK3-mutant rodents.

Increased self grooming and other repetitive behaviors have been consistently reported in the models ex4-22|ALL [128–130], ex11|SH3 [123], ex13|PDZ [139, 176], ex13-16|PDZ [83, 90, 155, 159, 160, 162–164, 166, 168, 181–183], ex14-16|PDZ [131], ex21|PRO [125, 178, 179], and ex11-21|SH3-PRO in rats [135]. Interestingly, in murine models ex21|PRO-InsG3680 [127] and ex8|ANK-Q321R [132], which mimick ASD-associated mutations, increased repetitive behavior was observed, whereas this was not the case in ex21|PRO-R1117X [127] mice, which harbor a schizophrenia-associated mutation. Additionally, cell-specific KO of SHANK3 in neocortical excitatory neurons, dorsal telencephalic excitatory neurons and glia, all or forebrain GABAergic neurons, and DRD2-positive neurons (ex14-16|PDZ-Emx1^Cre^, -Viaat^Cre^ [131, 133], ex4-22|ALL-NEX^Cre^, -Dlx5/6^Cre^, -Drd2^Cre^ [129]), but not in DRD1-positive neurons (ex4-22|ALL-Drd1^Cre^ [129]) was sufficient to induce repetitive behavior. Repetitive behavior was also described in mice of the model ex4-9|ANK, but findings were inconsistent across multiple studies [78, 80, 124, 155, 167]. Animals of the models ex9|ANK [82], ex17|PRM-S685I [94], ex21|PRO-InsG3728 [126], ex13-16|PDZ-Advillin^Cre^ [140], ex13-16|PDZ-Cdx2^Cre^, or ex6|ANK in rats [134] did not display abnormal repetitive behaviors.

Perseveratory behavior can be observed in tests that demand the reversal of a previously acquired behavior such as in the various maze tasks, primarily targeting cognitive abilities.

The models ex4-22|ALL [128, 130], ex11|SH3 [123], and ex21|PRO [125] show perseveration in the reversal part of these tasks, while the models ex13-16|PDZ [90, 155] and ex9|ANK [82] showed normal cognitive flexibility. Mixed evidence was reported in mice of the model ex4-9|ANK [78, 80, 124, 155, 167].

Lastly, restricted interests can be investigated in rodents by analyzing their reinvestigatory behavior or preference for certain objects, for instance in the hole board task or repetitive novel object task. However, such analysis has not been performed very frequently [78, 128–130].

Restricted investigatory behavior has been observed in the murine models ex4-22|ALL [128] and ex4-9|ANK [78]. Additionally, cell-specific SHANK3-KO in GABAergic neurons of the forebrain as established in mice of the model ex4-22|ALL-Dlx5/6^Cre^ was sufficient to induce this phenotype, whereas targeting neocortical excitatory neurons, DRD1-, or DRD2-positive neurons (ex4-22|ALL-NEX^Cre^, -Drd1^Cre^, -Drd2^Cre^) was not [129].

##### Additional behavioral phenotypes

ASD is frequently accompanied by additional behavioral phenotypes and comorbidities. Among these features are anxiety and hyperactivity [152, 184]. Avoidance behavior as observed in pathological demand avoidance (PDA) has also been proposed to be a subtype or symptom of ASD, while it was originally described as independent disorder [185]. A “comorbid” PDA has also been described in a patient harboring a frameshift variant within *SHANK3* [186].

In fact, one of the strongest phenotypes in SHANK3-deficient mice is an active avoidance behavior, which can be observed for instance in the marble burying test and novelty preference tasks, making it difficult to interpret those tests concerning their original targets. Such avoidance of inanimate objects could also influence the preferences observed in three-chambered tasks testing for sociability, where such objects are often placed in the “non-social” chamber.

Avoidance to novelty or inanimate objects was strong in two strains of the model ex4-22|ALL [128, 130], which also showed increased escape attempts, but was also consistently described in mice haboring other deletions or mutations such as ex9|ANK [82], ex11|SH3 [123], ex13-16|PDZ [155, 160, 163, 166], ex21|PRO [125, 178], and ex21|PRO-InsG3728 [126, 187]. It was also observed that targeted KO in somatosensory neurons or cells of the caudal embryo in mice of the strains ex13-16|PDZ-Advillin^Cre^ or -Cdx2^Cre^ induced such avoidance behavior [140]. Findings were inconsistent in the murine model ex13|PDZ [139, 176]. Avoidance behavior was absent in ex4-9|ANK [80, 124, 155, 167] or ex17|PRM-S685I [94] mice.

Anxiety-like behavior in rodents is often analyzed in paradigms like the elevated plus maze and dark-light emergence task or by thigmotaxis and rearing frequency in the open field. Increased anxiety has been observed in various, but not all murine models of SHANK3 deficiency.

High levels of anxiety were reported in models with the deletions ex4-22|ALL [128–130], ex13-16|PDZ [83, 90, 155, 159, 160, 163, 164, 168, 183, 188, 189], ex11-21|SH3-PRO in rats [135], and in mice with schizophrenia- or ASD-associated mutations (ex21|PRO-R1117X, -InsG3680 [127] or -InsG3728 [126]), while only slight differences were observed in the models ex13|PDZ [139, 176] and ex14-16|PDZ [131]. Elevated anxiety levels were also replicated in conditional KO-strategies, inducing SHANK3 deficiency in somatosensory neurons or cells derived from the caudal embryo (ex13-16|PDZ-Advillin^Cre^ or -Cdx2^Cre^ [140]), but also in GABAergic (ex14-16|PDZ-Viaat^Cre^ [131]) or DRD1-positive neurons (ex4-22|ALL-Drd1^Cre^ [129]). A minor anxious phenotype was observed in mice in which GABAergic neurons were targeted specifically in the forebrain (ex4-22|ALL-Dlx5/6^Cre^ [129]). Findings concerning anxiety-like behavior were not consistent in the models ex4-9|ANK [78, 80, 124, 155, 167] and ex21|PRO [125, 178], whereas no such behavior was reported for animals of the strains ex4-7|ANK [90], ex9|ANK [82], ex11|SH3 [123], ex6|ANK rats [134, 180], ex8|ANK-Q321R [132], ex17|PRM-S685I [94], ex14-16|PDZ-Emx1^Cre^ [133], and ex4-22|ALL-NEX^Cre^ or -Drd2^Cre^ [129].

Increased aggression was described in mice of the model ex11|SH3 [123].

Lastly, an altered circadian rhythm was reported in the murine model ex4-9|ANK [190], but not in ex11|SH3 [157] or ex13-16|PDZ [189].

#### Neurological phenotypes

##### Motor function

PMDS patients typically present with variable motor anomalies that persist beyond childhood, especially early-onset hypotonia with a resulting gait disorder, and impaired motor coordination [142, 152]. Accordingly, SHANK3 deficiency in rodents has commonly led to motor dysfunction across various domains, detected as abnormal sensorimotor or neuromuscular function, impaired motor learning, and altered general activity in rodents.

Sensorimotor function is often assessed in tasks such as the rotarod, which primarily targets coordination, by motor reflexes induced through sensory stimuli such as the righting reflex and geotaxis, and by gait analysis. Abnormalities concerning tasks depending on sensorimotor function have been described in SHANK3-deficient rodents of the deletion models ex4-22|ALL [128, 130], ex4-9|ANK [78, 124, 155, 167], ex11|SH3 [123], and ex13-16|PDZ [83, 155, 159, 183], or mice harboring schizophrenia- and ASD-associated mutations (ex21|PRO-R1117X, -InsG3680 [127] or -InsG3728 [126, 187]). Notably, single studies did not observe differences between wildtype and mutant mice of some of these strains [80, 90, 166]. Mixed evidence concerning sensorimotor dysfunction has been reported in the murine models ex13|PDZ [139, 176] and ex21|PRO [125, 178, 179]. No deficits were described in SHANK3-deficient rats (ex6|ANK [134], ex11-21|SH3-PRO [135]), in mice harbouring an ASD-associated mutation localized in a phosphorylation site relevant to the binding of ABI1 (ex17|PRM-S685I [94]), and if SHANK3 was specifically deleted in excitatory neocortical neurons, GABAergic neurons of the forebrain or DRD1-/DRD2-positive cells (ex4-22|ALL-NEX^Cre^, -Dlx5/6^Cre^, -Drd1^Cre^, -Drd2^Cre^ [129]).

Often sensorimotor testing in the accelerating rotarod is combined with an analysis of motor learning capability, which should result in an increasing score across multiple trials. Impaired motor learning was found in the deletion model targeting all isoforms of SHANK3 (ex4-22|ALL [130]), or in models mimicking mutations within exon 21 associated to neuropsychatric disorder (ex21|PRO-InsG3680, -R1117X, -InsG3728 [126, 127, 187]). In addition targeting neocortical excitatory neurons (ex4-22|ALL-NEX^Cre^) was sufficient to induce deficitary motor learning, while SHANK3 deficiency in GABAergic neurons of the forebrain, DRD1- or DRD2-positive neurons (ex4-22|ALL-Dlx5/6^Cre^, -Drd1^Cre^, -Drd2^Cre^) was not [129]. Divergent results across multiple studies were reported for the models ex4-9|ANK [78, 80, 124, 167] and ex13-16|PDZ [83, 90, 166, 183]. Motor learning skills were not affected in the murine models ex11|SH3 [123], ex13|PDZ [176], ex21|PRO [125, 178], and ex17|PRM-S685I [94].

Neuromuscular function has not been assessed frequently in SHANK3-deficient animals, but can be analyzed via simple methods utilizing a grip strength meter or in a hanging wire task. Interestingly, the only model in which consistent neuromuscular impairments were reported targets exon 11 for deletion (ex11|SH3), implying specific functions of SH3-domain containing isoforms in the skeletal muscle [111, 123]. However, neuromuscular dysfunction has also been reported in the deletion models ex4-22|ALL [130] and ex4-9|ANK [78], albeit inconsistently [124, 128, 167]. Intact neuromuscular function was reported in SHANK3-deficient rats of the model ex11-21|SH3-PRO [135].

Decreased locomotion in the open field is the most frequently used parameter to analyze the general motor activity of rodents. Hypoactivity has been observed quite frequently and consistently in various models of SHANK3 deficiency including the deletion models ex4-22|ALL [128–130], ex11|SH3 [157], ex13-16|PDZ [83, 155, 159–163, 168, 182, 183, 188, 189], ex13|PDZ [139, 176], ex14-16|PDZ [131], and ex21|PRO [125, 178, 191], as well as in models mimicking point mutations associated to ASD or schizophrenia (ex21|PRO-InsG3728, -InsG3680, -R1117X [126, 127, 187]). Decreased motor activity was reproduced by targeting all GABAergic neurons (ex14-16|PDZ-Viaat^Cre^ [131]), GABAergic forebrain neurons, DRD1-positive neurons (ex4-22|ALL-Dlx5/6^Cre^ or -Drd1^Cre^ [129]), or somatosensory neurons in homozygous mutant mice (ex13-16|PDZ-Advillin^Cre^ [140]). Notably, an increased locomotion was reported in ex4-22|ALL-NEX^Cre^ or -Drd2^Cre^ [129], suggesting opposing roles of SHANK3 in different types of neurons or divergent compensatory mechanisms depending on the affected circuitry. But this hypoactive phenotype was not always reported [90], even showing divergent results in the models ex4-9|ANK [78, 80, 124, 155, 167] and ex9|ANK [82, 175]. Other models with similar or different strategies to target the *Shank3*-gene, such as ex4-7|ANK [90], ex8|ANK-Q321R [132], ex17|PRM-S685I [94], ex13- 16|PDZ-Cdx2^Cre^ [140], ex14-16|PDZ-Emx1^Cre^ [133], or ex6|ANK [134, 180] and ex11-21|SH3-PRO in rats [135], did not show altered motor activity.

##### Sensory function

Hypersensitivity, hyposensitivity, or unusual interest in sensory stimuli is a common feature of ASD [3] and also PMDS patients, who often display an increased pain tolerance [152]. Thus, it is not surprising that abnormal sensory function has also been described in SHANK3-deficient rodents.

Sensory function can be analyzed in various modalities. The most frequently used paradigms focus on nociception or somatosensory function in general, which can be assessed via simple methods such as the tail flick test, certain reflexes, the Frey test, or the hot plate test. But also other modalities, including audition, olfaction, vision, vestibular function, and sensorimotor gating, have been investigated.

Abnormalities of somatosensory function in general, but mostly hyposensitivity to painful stimuli have been described in the models ex4-22|ALL [92, 130], ex11|SH3 [123], ex21|PRO [125], and ex11-21|SH3-PRO in rats [135]. Targeted disruption of SHANK3 in cells of the caudal embryo, somatosensory neurons (ex13-16|PDZ-Cdx2^Cre^, -Advillin^Cre^ [140]), and cells expressing SCN10A (also known as Nav1.8, ex4-22|ALL-Nav1.8^Cre^ [92]) was sufficient to induce somatosensory dysfunction. Notably, somatosensory dysfunction concerning light touch stimuli had been described by two studies examining the constitutive SHANK3-deficient model ex13-16|PDZ [159, 192], while in another study somatosensory function as examined by thermo-/nociception was not affected in these mice [163]. Mixed evidence was also reported for the murine model ex4-9|ANK [124, 140, 167], while somatosensory function was not affected in mice of ex8|ANK-Q321R [132].

Generally, other senses like vision [125, 126, 130, 166, 193], olfaction [124, 135, 161, 163, 176] or vestibular function [134, 155] were not affected in SHANK3-deficient rodents. Although one study using the model ex4-22|ALL found abnormalities in tasks depending on olfaction or vestibular function [130].

Audition is often assessed in parallel to the testing of sensorimotor gating in an auditory prepulse inhibition analysis. Baseline auditory startle responses were impaired in mice of the models ex4-22|ALL [129, 130], ex13-16|PDZ [155, 163, 166], and ex21|PRO-InsG3680 or -R1117X [127]. These findings were also replicated by specific targeting of GABAergic forebrain neurons in ex4-22|ALL-Dlx5/6^Cre^ mice [129], but not if SHANK3-deficiency was induced in neocortical excitatory neurons (ex4-22|ALL-NEX^Cre^ [129]), cells of the caudal embryo, or somatosensory neurons (ex13-16|PDZ-Cdx2^Cre^, -Advillin^Cre^ [140]). Audition was unaltered in the models ex4-9|ANK [78, 80, 124, 140, 155], ex13|PDZ [139], ex21|PRO [125], and ex17|PRM-S685I [94]. These mice did also display intact sensorimotor gating as measured by prepulse inhibition tests.

Sensorimotor gating was reported to be decreased in the models ex4-22|ALL [129], ex13-16|PDZ [155], and ex21|PRO-InsG3680 or -R1117X [127], although these findings could be influenced by the abovementioned auditory dysfunction. Additionally, some studies found no differences between wildtype and mutant mice of these models [130, 163, 166]. In single studies targeting of GABAergic forebrain neurons in ex4-22|ALL-Dlx5/6^Cre^ mice [129], cells of the caudal embryo, or somatosensory neurons (ex13-16|PDZ-Cdx2^Cre^, -Advillin^Cre^ [140]) was sufficient to disrupt sensorimotor gating, either in an auditory or tactile prepulse inhibition test.

##### Cognitive function

The majority of PMDS patients exhibit a severe or profound intellectual disability [152]. As a consequence, cognitive performance in SHANK3-deficient animals has been tested across multiple modalities, involving various brain regions for instance hippocampal or amygdala-dependent paradigms using contextual and cued fear conditioning as measures of associative learning, or spatial learning tasks in various maze configurations.

Basic recognition memory is often assessed by novel object recognition tasks, which require extensive pretesting to ensure the absence of confounding factors. Recognition memory was impaired in the models ex4-22|ALL [130], ex4-9|ANK [78, 80, 124, 140], ex11|SH3 [193], and ex11-21|SH3-PRO in rats [135]. Mice from the models ex8|ANK-Q321R [132], ex9|ANK [82], and ex13-16|PDZ or associated conditional KO animals [140, 163] were unaffected.

Decreased spatial learning was observed in ex4-22|ALL [128, 130], ex9|ANK [82], ex11|SH3 [123], ex21|PRO [125], and ex21|PRO-InsG3728 [126], but not in other models investigated, such as ex6|ANK rats [134], and mice harboring mutations associated to neuropsychiatric disorder (ex21|PRO-InsG3680 or -R1117X [127]). Divergent results were reported for ex4-9|ANK [78, 80, 124, 155, 167], ex13|PDZ [139, 176], and ex13-16|PDZ [90, 155, 162, 163, 166].

Associative learning was not consistently altered in any of the investigated exon specific or mutated models ex4-9|ANK [80, 124, 167], ex13|PDZ [176], ex13-16|PDZ [163], ex8|ANK-Q321R [132], or ex6|ANK in rats [134], while it was clearly affected in ex4-22|ALL mice [128–130]. These deficits were not induced by targeting neocortical excitatory neurons or GABAergic forebrain neurons in ex4-22|ALL-NEX^Cre^ or -Dlx5/6^Cre^ mice [129]. Interestingly, ex4-22|ALL SHANK3-deficient mice showed increased responses in the amygdala-dependent paradigm, but impaired associative learning in the hippocampus-dependent task in one study [130]. In a specific task testing for cerebellum-dependent function, heterozygous ex21|PRO mice also displayed deficient associative learning [194].

Striatal-dependent instrumental learning was severely impaired in the model ex4-22|ALL [128], suggesting the involvement of reward-related processes that are required in this task. These could potentially affect social reward circuitry. Together with abnormal functional (hyperactive) and socially induced (hypoactive) connectivity in the cortico-striatal-thalamic circuitry, the resulting ASD-like behaviors might be explainable by these changes [128].

Strong deficits in a paradigm examining perceptual learning were found in the model ex13-16|PDZ [188].

These differential results concerning the various domains of learning and memory could result from the varying expression patterns of SHANK3 isoforms in different brain regions. But these deficits have not been causally related to certain dysfunctional brain circuits.

Despite the fact that ADHD is a frequently described comorbidity of ASD [3], such deficits have seldomly been studied in animal models of SHANK3 deficiency. Impaired attention has only been observed and studied in the murine model ex11|SH3 [193] and the rat model ex6|ANK [134]. Such impairments should thus be reinvestigated in other models. Attention deficits could potentially influence many of the abovementioned behavioral and neurological phenotypes.

##### Additional neurological phenotypes

Since some patients with PMDS or SHANK3-associated ASD suffer from epilepsy [142, 195], it is of interest that such a phenotype has only been observed rarely in SHANK3-deficient mice [90]. On the contrary, some studies observed decreased susceptibility to PTZ-induced seizures in the models ex13-16|PDZ [163] and ex8|ANK-Q321R [132].

Furthermore, in the murine model ex21|PRO abnormal sleep patterns have been described [191], recapitulating a common cause of distress for both the PMDS patients themselves and also their parents [142].

#### Recapitulation of phenotypes in heterozygous animals

The sections above mainly focused on phenotypes observed in homozygous SHANK3-deficient rodents. Some studies reported that heterozygous SHANK3-deficient animals of various models were also affected, albeit less severely and also much less replicated than the phenotypes of homozygous mutants. Due to the fact that PMDS is a haploinsufficiency by definition, heterozygous animals should be studied more frequently, but might need more sensitive testing methods and bigger testing cohorts.

Abnormal social behavior was present in heterozygous mice from models targeting the ankyrin repeats (ex4-9|ANK [80, 122, 124]), the PDZ-domain (ex13-16|PDZ [140, 196], ex13|PDZ [139, 176]) or the prolin-rich region (ex21|PRO [154, 156, 177, 179, 197–199]), but also in heterozygous SHANK3-deficient rats (ex6|ANK [134, 180], ex11-21|SH3-PRO [135]) and models mimicking mutations associated to ASD or schizophrenia (ex8|ANK-Q321R [132], ex17|PRM-S685I [94], ex21|PRO-R1117X [127]).

Stereotypies were observed in heterozygous animals from multiple exon-specific models (ex4-9|ANK [80, 124], ex13-16|PDZ [140, 166], ex13|PDZ [139, 176], ex21|PRO [154, 156, 177, 199], ex11-21|SH3-PRO rat [135]), and in mice harboring an ASD-associated mutation affecting the ANK-domain (ex8|ANK-Q321R [132]).

Avoidance towards inanimate objects was described in heterozygous mice of two exon-specific SHANK3-mutant lines (ex13-16|PDZ [140], ex21|PRO [178]).

Concerning the replication of phenotypes representative of ASD comorbidities, heterozygous mutant rodents of some exon-specific models (ex4-9|ANK [167], ex13-16|PDZ [140], ex13|PDZ [139, 176], ex11-21|SH3-PRO rat [135]) or mice with SHANK3 point mutations (ex21|PRO-R1117X [127], ex21|PRO-InsG3680 [127]), which are associated to human neuropsychiatric disorder, exhibited increased anxiety-levels compared to wildtype animals. Aggressive behavior was only described in one study on heterozygous mice of the model ex21|PRO [198].

Finally, neurological symptoms reminiscent of deficient motor skills (ex4-22|ALL [130], ex4-9|ANK [167, 200], ex11|SH3 [123], ex13-16|PDZ [140], ex13|PDZ [139, 176], ex21|PRO [179], ex21|PRO-InsG3680 [127]), abnormal sensory processing (ex4-22|ALL [92, 130], ex13-16|PDZ [140, 201], ex11-21|SH3-PRO rat [135]) or impaired cognitive capabilities (ex4-9|ANK [80, 167], ex13-16|PDZ [140, 188, 201], ex21|PRO [194], ex6|ANK rat [134]) described in PMDS patients were also present in some of the investigated heterozygous rodent models of SHANK3 deficiency.

#### Structural and functional deficits

SHANK3-deficient animal models have been extensively investigated concerning disrupted structural or functional features primarily of the brain. Such changes have been observed on the molecular level, for instance as altered protein composition, protein localization, or transcription, but also in physiological processes, such as synaptic transmission or plasticity, and lastly as abnormal morphology.

##### Molecular changes

A great number of proteins is altered, when the *Shank3*-gene is disrupted. Most of them directly or indirectly interact with SHANK3 and are involved in the mediation of protein-protein interaction themselves, are associated to actin-associated processes or the cytoskeleton in general, or represent subclasses of glutamate receptors. Beside these main classes of affected proteins, a wide variety of other proteins and genes have been altered upon SHANK3 deficiency.

Studies have often focused on certain brain regions to analyze these defects, among which the striatum is the most frequently investigated and apparently also the most affected. Multiple proteins involved in the mediation of protein-protein interactions, especially HOMER1 [80, 90, 123, 128, 129, 139, 202], but also HOMER2 [128], HOMER-proteins in general [83, 127, 135], DLG2 [90, 127], DLG4 [80, 94, 127, 135, 139], DLGAP2 [202], and DLGAP3 [83, 90, 127, 128], were decreased in the striatum of mutant animals. Additionally, reduced levels of various glutamate receptor subunits, such as AMPAR-subunits (GRIA1 [135, 202], GRIA2 [80, 83, 90, 127, 139, 202, 203], GRIA3 [80, 139]), NMDAR-subunits (GRIN1 [127, 135, 139, 202, 203], GRIN2A [83, 90, 127, 139], GRIN2B [83, 90, 127, 139, 202]), GRM5 [127], and GRIK5 [202] were observed, mainly in synaptic fractions of the striatum. Another cluster of proteins that was decreased in striatal synapses of SHANK3-deficient animals is associated to actin or the cytoskeleton such as DOCK4 or TRIO [202]. Most interestingly, the other affected proteins of this group and more specifically WASF1 [94], NCKAP1 [202], ABI1 [94], or BAIAP2 [202] are either integral parts or regulators of the WAVE regulatory complex, which is crucial for the RAC1-dependent and ARP2/3-mediated actin polymerization [204]. Other proteins that were found to be decreased in striatal synapses or the striatum in general include the potassium channels KCNJ2 and KCNMA1 [202], the MECP2-associated kinase CDKL5 [202], GNAS [202], SYNGAP1 [127], CYLD [205], and the interneuron-marker PVALB [206]. Although most changes of protein levels showed a trend towards reduction, increased levels of the cell adhesion proteins CNTN2 and CDH4, MYEF2, which serves as a transcriptional repressor of the myelin basic protein, the Akt-regulating E3 ubiquitin-protein ligase TTC3, and members of the ribosomal protein family have been observed in striatal synapses [202]. Contradicting some of the abovementioned results, increased levels of GRIA1 [127] and GRM5 [128] have also been observed in some studies, which could be due to the different genetic targeting of *Shank3*.

Albeit synaptic protein composition was reported to be especially altered in the striatum, decreased levels of similar groups of proteins were also observed in the hippocampus, affecting HOMER1 [78, 128, 202], HOMER2 [128], HOMER3 [128, 202], GRIA1 [78, 122, 139, 203], GRIN1 [139], GRIN2A [128], and the actin- or cytoskeleton-associated proteins ABI1, PFN2, GSN, FARP2, and DOCK3 [202]. Other proteins that were shown to be reduced in hippocampal synapses include SYN3, CYLD, and proteins of the YWHA-family [202]. Furthermore, protein levels of the glutamate receptor subunits GRIN1 [129, 135], GRIN2B [118, 126, 129], and GRM5 [125], the calcium channel subunit CACNA1B, NLGN2, the cytoskeleton-associated kinase FER, the ribosomal protein RPL27A, and ALDH5A1 [202], which is involved in GABA-degradation, were increased in hippocampal synapses. Notably, one study reported increased synaptic DLG4-levels in heterozygous ex11-21|SH3-PRO rats [135].

Analogous to the findings in the striatum and hippocampus, proteins like HOMER1 [123] or HOMER in general [127], DLG2 [127], and DLG4 [127, 177], which are important mediators of protein-protein interactions, were found to be reduced in cortical synapses. Further proteins, which were dysregulated in the cortex, include GRM5 [123], the NMDAR-subunits GRIN1 and GRIN2B [127, 177], SYNGAP1 [127], MAPK [207], the actin-isoforms ACTB and ACTG1, but also actin-associated proteins like CFL1, LIMK1, CAPZB, EZR, ITPKA, and RAC1 [177].

Similarly, in prefrontal areas like the anterior cingulate cortex protein levels of HOMER1 [134], GRIA1, GRIA2 [160], GRIN1 [177, 198, 199], GRIN2A [177, 197–199], and GRIN2B [160, 199] were reduced. Additional studies described dysregulated levels of CTNNB1 as regulator of Wnt-signaling [154] and ARC as a major regulator of plasticity [156]. These changes were related to increased nuclear HDAC2 [154], EHMT1, and EHMT2 levels [156] in the prefrontal cortex (PFC), which are important epigenetic regulators of gene expression. An increased amount of HDAC2, EHMT1, and EHMT2 generally results in reduced expression of the genes, directly affected by their epigenetic modifications, although indirect effects could also lead to increased gene expression.

Specific analysis of the insular cortex revealed dysregulated markers of inhibitory neurons as increased levels of GAD2, and decreased levels of PVALB were observed. Interestingly, the WFA-positive perineural net surrounding PVALB-positive neurons was also affected [208].

Brain regions other than the abovementioned were not regularly investigated, but decreased levels of thalamic GRIA2 and GRIN1 [203], cerebellar GRIA1 [83], GRIN1 [135] and GRM5 [179], HCN1 in the spinal cord [140], or HCN1 and TRPV1 in dorsal root ganglia [92, 140] have been described in SHANK3-mutant mice. The analysis of whole brain samples additionally revealed generally reduced synaptic amounts of HOMER1 [78], DLGAP1 [78], GRIA1 [78], or GRIA-subunits in general [118], GRIN2A [78], and HCN2 [209].

A single study reported markedly increased levels of general protein synthesis activity in a multitude of brain region like the cortex, hippocampus, amygdala, raphe nucleus, thalamus, hypothalamus, cerebellum, and corpus callosum [207].

Notably, compensatory mechanisms among SHANK-proteins have been described in the striatum, hippocampus, and cortex. SHANK2 has been observed to be upregulated in whole brain lysates, the striatum [118] and cortex [83, 127]. In addition to the overexpression of remaining SHANK3-isoforms [132], the expression of previously undetected low-molecular SHANK3-isoforms has been reported in all three regions [125, 126, 151] of SHANK3-deficient rodents.

Some proteins, whose levels were altered in certain subcellular compartments, additionally displayed defective subcellular localization, induced by SHANK3 deficiency. Among these proteins are HOMER1 [78, 80, 123, 128, 139] and DLG4 [80, 135, 139] as protein-protein interaction mediators, the AMPAR- or NMDAR-subunits GRIA1 [135], GRIA2 [80, 139], GRIA3 [80, 139], GRIN1 [135, 139, 177, 198, 199], GRIN2A [139, 177, 198, 199] and GRIN2B [139, 199], the metabotropic glutamate receptor GRM5 [123, 125, 128], and TRPV1 [92]. Other dislocalized proteins, which are associated to actin or the cytoskeleton, include ABI1, WASF1 [94], and CTNNB1. CTNNB1 specifically localized less to synapses, but more to the nucleus, where it was found to be enriched on the promotors of *Hdac2* [154], *Ehmt1*, and *Ehmt2* [156], which might be the reason for their upregulation. Conversely, other studies found that a specific mutation can lead to sequestering of CTNNB1 by SHANK3 in nuclear bodies and thus decrease its functionality as transcriptional activator [105]. This divergent influence resulting from different forms of SHANK3 deficiency reflects the clinical heterogeneity, which can be observed in PMDS-patients.

Fitting their dysregulated subcellular localization, the interaction between HOMER1 and GRM5 was reduced by SHANK3 deficiency [123, 128]. Furthermore, an ASD-associated mutation of SHANK3 at a crucial phosphorylation site was sufficient to disrupt its interaction with ABI1 [94].

Matching the generally observed molecular alterations, SHANK3 deficiency resulted in impaired signaling mediated by metabotropic glutamate receptors (e.g., GRM5) [123, 128] and decreased phosphorylation of SYN1, CREB [210], and targets in the PI3K/AKT/MTOR- [178] or MAPK/ERK-pathway [92], also affecting synaptic MAPK-phosphorylation [106, 207]. Furthermore, the reduction of crucial components belonging to the RAC1-dependent signaling pathway, such as SHANK3-interactor ARHGEF7 or LIMK1, that leads to the phosporylation and thus inactivation of CFL1, might result in an increased actin-depolymerization by the active form of CFL1 [177]. Apart from dysregulated phosphorylation, abnormal S-nitrosylation of STX1A, PPP3C (also known as Calcineurin A), and several proteins involved in ASD-associated processes was recently reported [210].

Most studies have focused on the effects of SHANK3 deficiency on the protein level, but some alterations have also been observed with regard to transcription. Recent advances in RNA-sequencing techniques and their widespread use have enabled the unbiased transcriptomic analysis of certain brain regions. In SHANK3-deficient mice the PFC has so far been the only region investigated by this approach. Hundreds of genes were reported to be altered in these studies [154, 156, 191]. Fitting some of the proteomic changes described above, dysregulated transcripts were often associated to actin-associated processes, cell adhesion, signal transduction pathways and regulation of phosphorylation, developmental processes and cell morphogenesis, regulation of transcription, and regulation of protein stability [154, 156]. Many affected transcripts, such as *Homer1*, represented ASD susceptibility genes themselves [156]. Transcripts, which were additionally altered following sleep deprivation compared to baseline conditions, clustered in categories associated to MAPK/GnRH-signaling and the regulation of circadian rythmicity [191].

Validation of some of the altered genes by PCR showed that RNA levels of *Arc*, *Homer1* [156, 197, 198], *Grin1* [198], and *Sgk2* [197] were reduced in the PFC. Additionally, prefrontal *Hdac2*- [154], *Ehmt1*-, and *Ehmt2*-transcript levels [156] were increased, which was accompanied by epigenetic dysregulation. Transcripts of RAC1-associated pathways, such as *Arhgef7* [154, 199] and *Limk1* [154] were reduced, which fits the disrupted actin-cytoskeleton observed in the PFC [154, 177, 199] and hippocampus [177]. Other studies found striatal *Pvalb*-transcripts [206] and hippocampal *Gabra1*-, *Gabra2*-, or *Gabrb1*-transcripts of GABA A receptor subunits [211] to be reduced. Importantly, a compensatory increase of *Shank1*, *Shank2* [127], and transcripts of non-deleted *Shank3*-exons [151] was observed.

Other major factors influencing gene expression in vivo are epigenetic regulatory mechanisms. Fitting the above mentioned dysregulation of HDAC2, EHMT1, and EHMT2, a decreased H3-acetylation and increased levels of H3K9m2 di-methylation especially at the *Arc* promotor region were observed in the PFC, but not other brain regions of SHANK3-deficient animals [154, 156, 198, 199]. Besides its pathophysiological importance, these findings pointing at epigenetic dysregulation could represent a promising therapeutic target, as epigenetic interventions could normalize expression levels of a multitude of dysregulated genes at once.

Although the majority of studies focus on alterations in the nervous system, dysregulated cytokine levels in SHANK3-deficient mice were recently reported [211]. Another peripheral organ, which seems to be affected by SHANK3 deficiency is the gastrointestinal system, as it was observed that TJP1 (also known as ZO-1) was increased in the small intestine, which might affect intestinal barrier function [212]. Additionally, increased bacterial lipopolysaccharide levels were found in the liver, suggesting an increased leakiness of the gastrointestinal system in these mice [212].

##### Alterations of physiological processes

Aside from molecular changes, the resulting dysfunction of physiological processes has been investigated in depth across multiple studies, focusing on crucial neuronal functions, such as synaptic transmission or plasticity. Although it has to be noted that not all abnormalities, which have been described, were reproduced consistently. However, this section will focus on studies that found physiological deficits in SHANK3-deficient animals.

Firstly, basic cellular excitability has been reported to be abnormal in striatal MSNs [128, 129, 182], hippocampal neurons [132, 213], prefrontal neurons [133, 160], thalamic neurons [209], and neurons of lumbar dorsal root ganglia [140].

Measures related to synaptic transmission, which are often dependent on NMDAR or AMPAR functionality, were deficient in the striatum [80, 83, 90, 94, 127, 128, 131, 139, 164, 168, 182, 214], hippocampus [82, 94, 122, 124–126, 129, 135, 177, 187, 200], PFC [82, 127, 133, 154, 156, 160, 177, 197–199], and thalamus [209], but also in the somatosensory and visual cortex [140], ventral tegmental area (VTA) [161], and spinal cord [92]. The study of the temporal development of measures related to synaptic transmission has also revealed that synaptic maturation is dysregulated in striatal MSNs [214]. Initially, SHANK3 deficiency resulted in a premature corticostriatal hyperactivity in early developmental stages [214], while the physiological rise of activity was insufficient, which led to depressed striatal activity during adulthood [90, 182, 214]. Interestingly, another study showed that corticostriatal hyperactivity results in different striatal activity patterns in adult mice, when induced at different developmental timepoints. Adult striatal activity was depressed when corticostriatal hyperactivity was induced early-on, which resembles the phenotype of SHANK3 deficiency, whereas it resulted in persistent hyperactivity when induced at later developmental stages [183].

Similar brain regions have been found to exhibit impaired mechanisms of synaptic plasticity, namely the striatum [123, 127, 128, 164, 182], hippocampus [78, 80, 122, 124–126, 134, 135, 139, 200], PFC [134, 160], VTA [161], and spinal cord [92]. A specific form of homeostatic plasticity, which also occurs upon sensory deprivation, was reported to be dysfunctional in the visual cortex of SHANK3-deficient mice [181].

Some studies have further investigated network activity and connectivity in certain neuronal circuits during SHANK3-deficient conditions. Abnormal baseline neuronal activity was observed in the cortex as a whole by EEG-measurement [132, 163] or by MEA in primary cortical cultures [215]. Specific brain regions were also analyzed by different methods revealing altered baseline activity in the auditory cortex [216], somatosensory cortex [192], hippocampus, striatum, and prefrontal areas [175], while neuronal activity induced by different types of social interaction was abnormal in the PFC [160, 175, 196], striatum, periaqueductal gray, lateral and medial habenulae, and lateral septum [175]. In addition to an abnormal baseline activity, neuronal activity induced by stimulating the vibrissae was altered in the somatosensory cortex, where hypoactive inhibitory interneurons led to a hyperactivity of excitatory pyramidal neurons [192]. Similarly, an abnormal GABAergic circuitry in the insular cortex led to impaired activity induction via audio-tactile stimuli and deficient multisensory integration [208]. Notably, neuronal activity induced by light touch stimuli was dysregulated in circuits of lumbar dorsal root ganglia [140]. Induced neuronal activity patterns were also disrupted in primary neuronal cultures derived from cortices of SHANK3-deficient mice [215]. Abnormal responses of neuronal circuits to speech sounds were reported in the auditory cortex of SHANK3-deficient rats [216].

Via activity measurements in the PFC during a paired interaction task, it was recently observed that the number of neurons encoding self-experience was increased, while those encoding the other social agent’s experience were decreased in SHANK3-deficient mice. Furthermore, there was no differentiation between self- and other-encoding neurons since all other-encoding neurons also responded to self-stimuli. Such a loss of differential neuronal encoding concerning social and other stimuli might be an essential element underlying the behavioral phenotypes of SHANK3-deficient animals [196].

Altered connectivity measures were reported in the cortico-striatal-thalamic circuitry, where an increased baseline functional connectivity between the nucleus accumbens and cingulate cortex or thalamus hindered a sufficient rise of coherence upon social induction of functional connectivity, which could be due to the altered excitability of striatal MSNs mentioned above [128]. Cortico-striatal connectivity was also found to be altered in independent studies, which used different methodologies [158, 214]. Furthermore, periodic synchronized activity patterns, as measure of connectivity in primary cultures, were impaired in cortical neurons of SHANK3-deficient mice [215]. Additional brain regions that showed dysregulated connectivity in an fMRI study included the hippocampus, claustrum, regions of the basal forebrain, and many cortical subareas (entorhinal, perirhinal, piriform, retrosplenial, motor, visual, and auditory cortex) [158].

Interestingly, physiological alterations that could hint at inflammatory processes in the cortex, such as astrogliosis and elevated IL6-levels, were reported in SHANK3-deficient mice [212]. Furthermore, signs of nitrosative stress were observed in the PFC and striatum [210].

Studies that also investigated non-brain associated phenotypes found a dysregulated microbiome composition in SHANK3-deficient mice, which was targeted to influence behavioral phenotypes [161, 211, 212].

##### Morphological abnormalities

Changes of the molecular composition and physiology in a tissue are often accompanied by morphological abnormalities, which have also been described especially in the brain of SHANK3-deficient animals. Again most of those anomalies have been observed in the striatum, hippocampus, and cortical areas. But this might also be due to the fact that those regions are the ones which many studies relied on in their analysis.

Abnormal dendritic spine or synapse morphology and an aberrant configuration of the dendritic tree in general have been observed in the striatum [83, 90, 94, 128, 182], hippocampus [78, 94, 122, 135, 213], regions of the PFC [127, 160, 217], and cerebellum [194]. Ultrastructural analysis additionally revealed an altered PSD morphology in the striatum [90, 128], hippocampus [218], and anterior cingulate cortex as part of the PFC [160].

Volumetric analysis by MRI techniques indicated an increased size of the striatum [90, 128], pallidum [128, 219], pretectum, superior colliculus, deep mesencephalic nuclei, brainstem, and tegmental areas, such as the substantia nigra and interpeduncular nucleus [128]. Conversely, the hippocampus [219], PFC [158], auditory cortex [158], retrosplenial cortex [158], olfactory areas [128], and the brain as a whole [219, 220] showed a decreased volume, while mixed evidence was reported concerning the thalamus [128, 219]. In addition the size of fiber tracts or their white matter integrity was reduced in the optic tract, fornix, fimbria, stria terminalis, anterior commissure, cerebral peduncle, spinal trigeminal tract, and cingulum of SHANK3-deficient mice [128].

General neuroanatomical examination of the somatosensory cortex, visual cortex, and amygdala uncovered a loss of PVALB-positive interneurons in these regions [140]. Additionally, the number of Purkinje cells in the cerebellum of SHANK3-deficient mice was reported to be reduced [179]. Other studies focusing on the hypothalamus and spinal cord found a reduction of oxytocin-positive hypothalamic neurons [161] and altered morphological properties of lumbar ventral horn motoneurons [111], which is a finding of great importance, considering that muscular hypotonia represents a hallmark feature of the PMDS. In-depth analysis of skeletal muscle tissue found an impaired maturation and complexity of neuromuscular junctions, as well as altered ultrastructural properties, especially a decreased Z-disc width in samples from ex11|SH3 mice and also PMDS-patients [111].

Another non-brain associated morphological abnormality was found in the small intestine of the murine model ex11|SH3, where the total villi length and ratio of villi length to crypt depth were decreased [212]. Also referring to the abovementioned observation of an altered microbiome [161, 211, 212], the gastrointestinal system might be an interesting peripheral target for further investigation. Since ASD and also the PMDS are frequently associated with gastrointestinal alterations [221–223], and the insular cortex, which has often been described as visceroceptive hub and center of multisensory integration in general [208, 224, 225], is also affected in SHANK3-deficient mice, dysregulated visceral signals to the insula or other involved regions might contribute to the overall phenotype by affecting the gut-brain axis [208].

#### SHANK3 deficiency in non-human primates

Recently, the CRISPR-Cas9-mediated generation of germline-transmissible mutations in exon 21 of *SHANK3* in cynomolgus macaques (*Macaca fascicularis*) and their F1 offspring was reported [136]. Although the founder animals represented a mixed cohort with non-uniform mutations, they were additionally tested for structural and behavioral abnormalities. MRI-based analysis revealed abnormal connectivity in the PFC, motor cortex, cingulate cortex, thalamus and striatum. Additionally, grey matter volume was generally reduced. Upon behavioral and neurological examination, founder mutants exhibited abnormal social behavior, increased stereotyped behaviors, motor deficits, hypoactivity, attentional deficits, learning impairments, and sleep disturbances [136]. Interestingly, a delayed pupillary light reflex was observed. Abnormal pupillary light reflexes have also been reported in ASD-patients [226].

Another group targeted exons 6 and 12 by CRISPR-mediated intervention in macaques [137, 227]. Generally, a lower pregnancy rate was observed and of the three reported pregnancies only one animal was born and survived carrying a mutation in exon 12. The surviving animal was thus examined by PET-MRI and behavioral analysis [227], while an aborted animal carrying mutations in both exon 6 and 12 was used for molecular and morphological analysis [137]. A general health screening demonstrated a decreased body weight and length, as well as an enamel dysplasia in the surviving individual. Behavioral analysis revealed abnormal social interaction and communication, repetitive behaviors, increased levels of anxiety, and exploratory hypoactivity. PET-MRI analysis showed a decreased baseline activity in the cortex, PFC, hippocampus, amygdala, and striatum [227]. On the molecular level, protein amounts of GRIN2B, DLG4, and RBFOX3 (also known as NeuN) were decreased in the PFC, while levels of GFAP were markedly increased. Similarly, in the striatum decreased levels of GRIN2B, DLG4, and RBFOX3, but also GRM5 and DCX were observed. Protein levels of GFAP were also increased in the striatum. Although total levels of HOMER1 were unaffected, it showed an abnormal localization to the cytoplasm in the PFC. Additional morphological analysis revealed a decreased spine density, and a reduced overall percentage and soma size of RBFOX3-positive cells in the PFC, while the proportion of GFAP-positive cells was elevated. These neuroanatomical findings together with the altered protein levels of RBFOX3 and DCX as a neural progenitor marker, suggested a heavily disrupted neurogenesis in this SHANK3-deficient individual [137], which had not been described in murine models.

#### Multiple knockout of Shank-genes

Earlier this year, the first double KO-model of SHANK1 and SHANK3 (SHANK1 +SHANK3-ex11|SH3) was published [138]. Generally, these mutant mice exhibited a markedly increased mortality, were generally smaller, and brain size was also reduced. On a molecular level these animals exhibited abnormal hippocampal and cortical synaptic protein composition and signaling related to AKT, MAPK, RPS6, and EEF2. Morphologically, both in the cortex and hippocampus a decreased spine density, an abnormal dendritic tree, and impaired PSD structure were observed. In hippocampal slices both excitatory synaptic transmission and plasticity were affected by SHANK deficiency. General cortical activity as measured by EEG was increased in SHANK-mutant animals. On a behavioral level these mice exhibited typical ASD-associated phenotypes. SHANK1 and SHANK3 deficiency resulted in impaired social behavior, increased repetitive, avoidance and perseverative behavior, abnormal spatial learning and recognition memory, as well as decreased sensorimotor capabilities and motor learning. Surprisingly, these animals showed lower levels of anxiety.

#### SHANK3-overexpression model

In contrast to the approach of deleting *Shank3* to study its biological function and role in human diseases, *Shank3*-EGFP transgenic mice overexpress all major SHANK3 isoforms and exons [93, 151]. These animals are characterized by a hyperkinetic phenotype that resembles mania. Their hyperactivity was aggravated after injection of amphetamine. Transgenic mice also displayed increased escape behavior, elevated sensorimotor gating, abnormal circadian rhythms, decreased social interactions, seizures, and hyperphagia-like behavior [93]. On a molecular level, SHANK3-transgenic mice habored elevated levels of F-actin [93, 228] and an increased spine density, which might be due to SHANK3’s influence on its interactors ARPC2 and WASF1, which are both part of the ARP2/3-complex and thus promote actin polymerization [93]. Additionally, the density of excitatory synapses was increased, while it was decreased for inhibitory synapses. This might be due to redistribution of the actin associated proteins ENAH and PFN2 from inhibitory to excitatory synapses. Both are connected to the SHANK3-interactome via WASF1 and ABI1, but are direct interaction partners of GPHN, a scaffolding protein of inhibitory synapses. Accordingly, excitatory synaptic transmission was increased, while inhibitory transmission was decreased [93]. Additional proteomic analysis revealed more than a hundred dysregulated proteins in striatal PSDs. Interestingly, the only proteins, which were also affected by SHANK3 deficiency rather than overexpression, were HOMER1, CYLD, SPATA2L, and RPL36A [202, 205]. The dysregulation of ribosomal proteins was further validated and increased levels of RPLP1 and RPL36A were observed in striatal synapses, but not in whole cell lysates. Generally, translation in striatal synaptosomes was elevated. With respect to signaling, MAPK-phosphorylation was increased [229], while MTOR-phosphorylation was decreased [228] in the striatum of SHANK3-overexpressing mice. This model was also extensively analyzed by RNA-sequencing in different brain regions, which revealed differential alterations of transcripts. Striatal samples showed changes in categories related to signaling pathways, phosphorylation, cellular metabolism, the ribosome, and others [228, 230]. Furthermore, specific investigation of polysome-associated transcripts showed increased levels of *Drd1* and its downstream target *Ppp1r1b* [229]. Conversely, in the PFC altered transcripts accumulated in categories associated to synaptic plasticity, the extracellular matrix, the ribosome and spliceosome, and myelination. Alterations related to myelin were validated by PCR and showed decreased levels of *Mobp*, *Mbp*, *Myrf*, *Mog*, and others [230]. Genes, which were found to be increased in the hypothalamus, clustered in categories associated to the ribosome and extracellular matrix, while decreased genes were associated to the synapse, microtubules, or axonal processes [231]. The only altered genes, which were commonly affected across the striatum, PFC, and hypothalamus were *Smim30*, *Gpr85*, *Cav2*, and *Kif5a* [228, 230, 231]. *Gpr85* RNA-levels were additionally increased in the hippocampus, cerebellum, and cortex [230]. Notably, Myelin-related transcripts were only decreased in the PFC, but not in the striatum or hippocampus [230]. Concerning treatment options, transgenic mice responded well to the anticonvulsant drug valproate, which ameliorated the hyperacitve and manic-like behaviors but did not influence the observed molecular phenotype [93].

### Treatment strategies for SHANK3 deficiency and their influence on subphenotypes in animal models

#### Pharmaceutical compounds

**Fig. 2:**
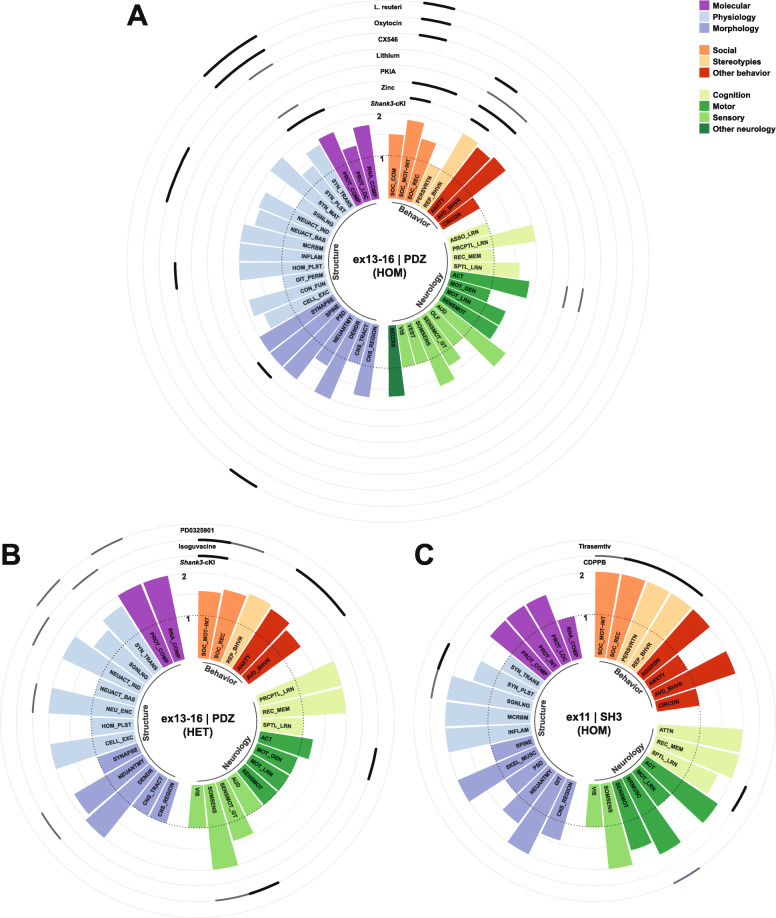
Phenotypes and effective compounds in the models ex13-16|PDZ and ex11|SH3. The phenotypes of the respective models are summarized according to the categories and interpretations, which were assigned to the performed experiments. On the *y*-axis the abnormality score (1 = normal; 2 = abnormal) is plotted per interpretation on the *x*-axis. Concentric circles are added around the circular barplot to illustrate the influence of a subset of treatment strategies on the phenotypes. Bold black lines indicate a full rescue of the observed deficits, while thinner grey lines indicate a partial normalization of the respective phenotype. Importantly, only beneficial effects were included in this visualization. The abbreviations used in this illustration are summarized in Table 1

**Fig. 3:**
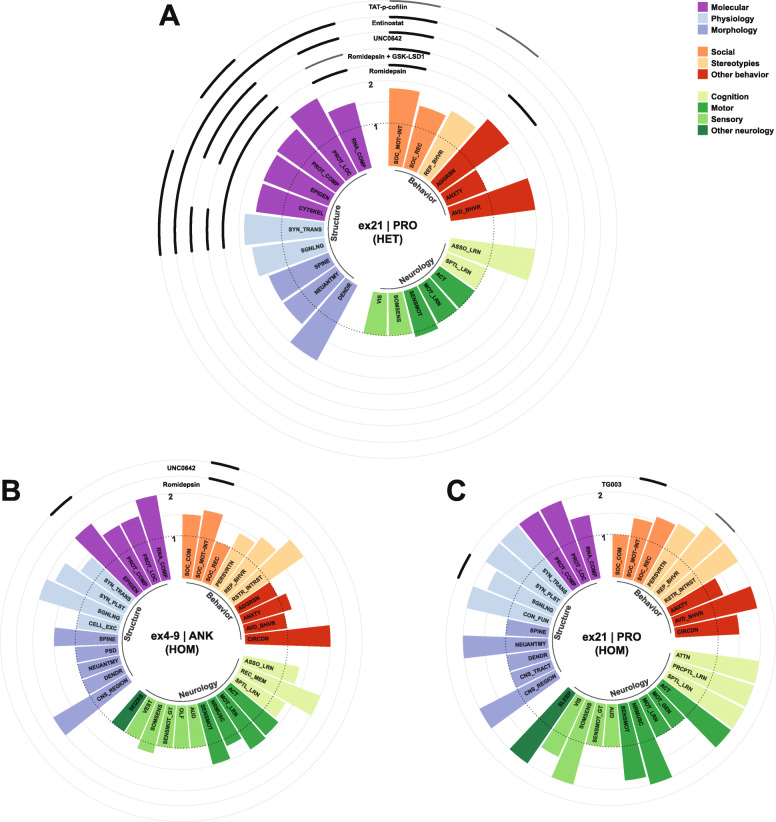
Phenotypes and effective compounds in the models ex21|PRO and ex4-9|ANK. The phenotypes of the respective models are summarized according to the categories and interpretations, which were assigned to the performed experiments. On the y-axis the abnormality score (1 = normal; 2 = abnormal) is plotted per interpretation on the x-axis. Concentric circles are added around the circular barplot to illustrate the influence of a subset of treatment strategies on the phenotypes. Bold black lines indicate a full rescue of the observed deficits, while thinner grey lines indicate a partial normalization of the respective phenotype. Importantly, only beneficial effects were included in this visualization. The abbreviations used in this illustration are summarized in Table 1

**Fig. 4:**
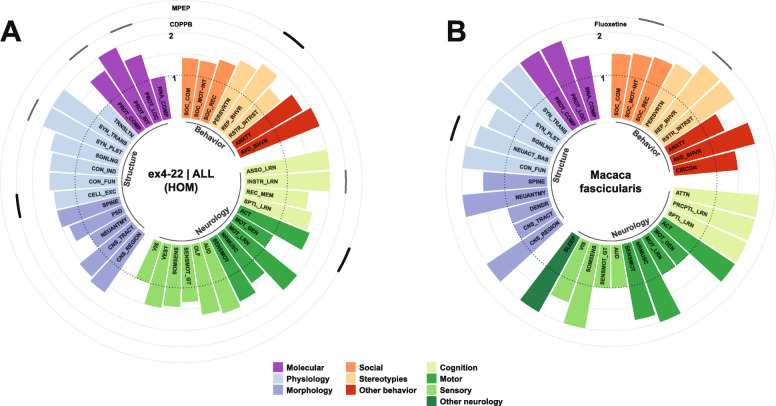
Phenotypes and effective compounds in the murine model ex4-22|ALL and SHANK3-deficient macaques. The phenotypes of the respective models are summarized according to the categories and interpretations, which were assigned to the performed experiments. On the *y*-axis the abnormality score (1 = normal; 2 = abnormal) is plotted per interpretation on the *x*-axis. Concentric circles are added around the circular barplot to illustrate the influence of a subset of treatment strategies on the phenotypes. Bold black lines indicate a full rescue of the observed deficits, while thinner grey lines indicate a partial normalization of the respective phenotype. Importantly, only beneficial effects were included in this visualization. The abbreviations used in this illustration are summarized in Table 1

Based on the abovementioned molecular and physiological abnormalities in SHANK3-deficient animals, multiple pharmaceutical compounds have been proposed as possible treatment options for SHANK3-associated neuropsychiatric disorder. For instance, it has been hypothesized that modulation of glutamate receptors might be beneficial, since AMPAR-, NMDAR-, or GRM5-hypofunction potentially leads to an excitation/inhibition imbalance, which could contribute to some ASD-like phenotypes in SHANK3-mutant mice [123, 128, 160, 162]. But many other strategies to target SHANK3-associated pathophysiology have been investigated. A subset of those treatment strategies is also visualized together with the phenotype observed in the respective model in Figs. 2, 3, and 4 (Table 1).

**Table 1:** Categories used in the database and circular barplots. Every data point was assigned to a category, subcategory, and an interpretation

Category	Subcategory	Interpretation	Shortcut
Structure	Molecular	Protein_composition	PROT_COMP
		Protein_localization	PROT_LOC
		Protein_interaction	PROT_INT
		RNA_composition	RNA_COMP
		Cytoskeleton	CYTSKEL
		Epigenetics	EPIGEN
	Physiology	Synaptic_transmission	SYN_TRANS
		Synaptic_plasticity	SYN_PLST
		Homeostatic_plasticity	HOM_PLST
		Cellular_excitability	CELL_EXC
		Signaling	SGNLNG
		Translation	TRNSLTN
		Functional_connectivity	CON_FUN
		Induced_connectivity	CON_IND
		Baseline_neuronal_activity	NEUACT_BAS
		Induced_neuronal_activity	NEUACT_IND
		Neuronal_encoding	NEU_ENC
		Synaptic_maturation	SYN_MAT
		Inflammation	INFLAM
		Microbiome	MCRBM
		GIT_permeability	GIT_PERM
	Morphology	Region	CNS_REGION
		Tracts	CNS_TRACT
		Neuroanatomy	NEUANTMY
		Spines	SPINE
		Synapses	SYNAPSE
		Dendrites	DENDR
		PSD	PSD
		Skeletal_muscle	SKEL_MUSC
		GIT	GIT
Behavior	Social	Social_motivationinteraction	SOC_MOTINT
		Social_recognition	SOC_REC
		Social_communication	SOC_COM
	Stereotypies	Repetitive_behavior	REP_BHVR
		Perseverative_behavior	PERSVRTN
		Restricted_interests	RSTR_INTRST
	Other_behavior	Anxiety	ANXTY
		Avoidance_behavior	AVD_BHVR
		Aggression	AGGRSN
		Escape_behavior	ESC_BHVR
		Food_intake	FOOD_INT
		Circadian_rhythm	CIRCDN
Neurology	Cognition	Spatial_learning	SPTL_LRN
		Associative_learning	ASSO_LRN
		Instrumental_learning	INSTR_LRN
		Perceptual_learning	PRCPTL_LRN
		Recognition_memory	REC_MEM
		Attention	ATTN
	Motor	Activity	ACT
		Neuromuscular	NRMUSC
		Sensorimotor	SENSMOT
		Motor_learning	MOT_LRN
		Motor_general	MOT_GEN
	Sensory	Somatosensory	SOMSENS
		Sensorimotor_gating	SENSMOT_GT
		Olfaction	OLF
		Vision	VIS
		Audition	AUD
		Vestibular	VEST
	Other_neurology	Seizures	SEIZRS
		Sleep	SLEEP

##### AMPAR-PAM

Positive allosteric modulation (PAM) of the AMPAR via the compound CX546 normalized social deficits of ex13-16|PDZ mice in one study [160], but had no effect on social behavior in another study [162]. Furthermore, AMPAR-PAM attenuated increased repetitive behaviors during social interaction [162] and partly corrected alterations of synaptic transmission in the anterior cingulate cortex [160]. CX546 did not affect the hypoactivity observed in ex13-16|PDZ mice [162].

##### NMDAR-agonism

Treatment of ex13-16|PDZ mutant mice with a partial agonist at the glycine modulatory site of the NMDAR (D-cycloserine) enhanced social interaction behavior, but had no effect on increased repetitive behavior or hypoactivity. Notably, a high dose of D-cycloserine induced hyperactivity in these animals [162].

##### GRM5-targeting

Different approaches to manipulate GRM5 signaling differentially influenced stereotypies and learning deficiency in ex4-22|ALL mice. While a GRM5-antagonist (MPEP) normalized hypoactivity and repetitive behaviors in these animals, a GRM5-PAM (CDPPB) improved striatal-dependent instrumental learning, cellular excitability and synaptic plasticity of striatal MSNs, and low synaptic levels of HOMER1. Notably, CDPPB worsened repetitive behavior in ex4-22|ALL mice [128], while an attenuated phenotype was observed in the murine model ex11|SH3 [123]. Behaviorally, positive allosteric modulation of GRM5 also resulted in an improved social interaction and recognition, normalized spatial learning, and reduced perseverative reversal-deficits in ex11|SH3 mice. Moreover, treatment with CDPPB ameliorated the deficient Calcium-signaling in cortical neurons and normalized GRM5-dependent NMDAR-functionality in striatal MSNs of this model [123].

##### GABAAR-agonism

Acute treatment with isoguvacine, a peripherally restricted GABAAR agonist that acts directly on mechanosensory neurons, reduced tactile hyperreactivity in six distinct ASD models, including ex13-16|PDZ SHANK3-deficient mice. Chronic treatment of SHANK3-mutant mice additionally improved body condition, brain abnormalities in the somatosensory cortex, anxiety-like behaviors, social deficits, avoidance behavior, and hypoactivity. Memory impairments and repetitive behaviors were not alleviated [140].

##### Nicotinic acetylcholine receptor (CHRN) PAM

The effects of chronic treatment with the PAM of nicotine receptors cotinine during SHANK-deficient conditions was studied in mice of the double-KO model SHANK1 +SHANK3-ex11|SH3. Prolonged treatment with cotinine normalized AKT-associated signaling, synaptic plasticity deficits, as well as spine density and dendritic complexity. Behaviorally, cotinine treatment rescued deficits of social behavior and recognition memory. Intriguingly, mortality was lower during treatment. Effects were not long-lasting and phenotypes regressed after the treatment was stopped [138].

##### MAP2K/MEK-inhibition

As it was shown that SHANK3 stability is reduced by activation of the MAPK/ERK-pathway, MAP2K/MEK-inhibition was further explored as possible therapeutic strategy. Indeed it was observed that chronic treatment of WT-mice or SHANK3-deficient animals with PD0325901, a selective, highly potent, and long-lasting MAP2K/MEK-inhibitor, resulted in an increased abundance of SHANK3 in cortical synapses and normalized phosphorylation ratios of MAPK1 in mutant mice. Similar effects were observed, when Pimasertib or Selumetinib, both MAP2K/MEK-inhibitors, were applied in a primary cell culture system of cortical neurons [106].

##### Oxytocin (OXT)

Due to its role as a major regulator of mammalian social behavior, OXT has long been proposed as possible point of convergence regarding the social deficits observed in ASD-patients [232]. It has been reported recently that OXT-treatment in ex13-16|PDZ mice rescued social interaction deficits and normalized induced synaptic transmission and plasticity in the VTA, while hypoactivity was not influenced. Notably, the beneficial effects of L. reuteri, which are further discussed below, were dependent on OXTR-signaling [161]. Furthermore, administration of OXT into the left lateral ventricle restored hippocampal synaptic plasticity and alleviated behavioral deficits, such as the impaired long-term social recognition memory and reduced attention levels in the ex6|ANK SHANK3-deficient rat model for PMDS [134].

##### IGF1

IGF1 or an active recombinant peptide (rhIGF1) alleviated deficitary synaptic plasticity and transmission, as well as motor deficits in heterozygous animals of the SHANK3 deficiency model ex4-9|ANK [200]. Additionally, deficits of neuronal morphology and excitatory neurotransmission in SHANK3-deficient rodent neurons and PMDS patient-derived neurons were also improved by application of IGF1 [178].

##### Lithium (Li)

Treatment with Li normalized the deficient mechanisms of homeostatic plasticity in the visual cortex and alleviated the overgrooming behavior in ex13-16|PDZ SHANK3-deficient mice. A similar disruption of homeostatic plasticity was also inducible via local RNAi-mediated knockdown of SHANK3 in the visual cortex, which was also restored either by Li- or GSK3-inhibitor-treatment [181]. In another study Li-application resulted in increased SHANK3-levels and rescued the maturation of neuromuscular junctions in a motoneuron-myotube coculture established from SHANK3-deficient PMDS-derived hiPSCs [111].

##### Fluoxetine

Fluoxetine, which is a well known so-called selective serotonin reuptake inhibitor (SSRI) and widely used as an antidepressant drug, was tested in a SHANK3-deficient macaque. After treatment, the individual showed improved social behavior including previously impaired active or passive social interaction and eye contact, alleviated repetitive behaviors, and normalized brain activity as assessed by PET-MRI.

##### Targeting of epigenetic mechanisms

Chromatin modifications, such as DNA-methylation, histone-methylation or -acetylation, but also histone-acylation, -homocysteinylation, -serotonylation, and others are crucial regulators of gene expression, summarized under the term epigenetics [233]. Recently, evidence has accumulated, which suggests that epigenetic mechanisms are dysregulated in SHANK3-deficient animals and thus enzymes that regulate histone-modifications were explored as therapeutic targets. Multiple studies reported a HDAC dysregulation resulting in decreased H3-acetylation levels in the PFC of mice from the models ex21|PRO [154, 198] and ex4-9|ANK [154], which was induced by an increased nuclear localization of the SHANK3-interactor CTNNB1 to the HDAC2 promoter region [154]. This dysregulation of epigenetic control mechanisms might affect actin-associated signaling like the RAC1-PAK-LIMK1-CFL1 pathway [154].

Because other findings led to the conclusion that these epigenetic changes linked to SHANK3 deficiency might underlie the social deficits in mutant mice, a brief treatment with romidepsin, a highly potent class I histone deacetylase (HDAC) inhibitor, was chosen as a therapeutic. Romidepsin alleviated social deficits in SHANK3-deficient mice in both models investigated (ex21|PRO [154, 198] and ex4-9|ANK [154]). The juvenile to late-adolescent period was reported to be the most effective therapeutic window. The treatment had no effect on increased repetitive behaviors [154]. On a molecular basis, HDAC2-inhibition elevated the expression and histone acetylation of *Grin2a* and actin-regulatory genes, for instance from the RAC1-pathway. This led to restored NMDAR-functionality and F-actin levels in the PFC of SHANK3-deficient mice. RNA sequencing data additionally showed that the majority of genes downregulated in SHANK3-deficient conditions (enriched in actin cytoskeleton-mediated transport, signal transduction pathways and developmental processes) were normalized following romidepsin treatment. Some genes identified as key ASD risk factors were increased after treatment, which might also be a potential mechanism for the romidepsin-induced rescue of social deficits [154]. An array of additional pharmacological agents currently used in psychiatric practice was tested, of which only valproic acid (mood stabilizer and low-affinity class-I HDAC inhibitor) showed some effect on social deficits in SHANK3-deficient mice, which were not long-lasting. This did also hold true for the pan-HDAC inhibitor Trichostatin A [154]. In an attempt to increase the durability of the effects of Romidepsin even in adult SHANK3-deficient mice, it was combined with GSK-LSD1, which selectively inhibits the histone demethylase KDM1A (also known as LSD1). Indeed this combination of compounds induced a long-lasting normalization of social behavior, aggression levels, synaptic protein levels, synaptic transmission, and histone acetylation even in adult animals for up to 21 days post-treatment. RNA-levels of *Grin1* were also partly normalized. Monotherapy with GSK-LSD1 did not induce a long-lasting amelioration of phenotypes [198].

Another promising candidate to target epigenetic mechanisms seems to be Entinostat (MS-275), a potent, long-lasting and PFC-selective class I HDAC-inhibitor. Treatment with Entinostat resued social behavior for up to 11 days post-treatment. Additionally, protein-levels of the NMDAR-subunits GRIN1/2A/2B on the cellular surface, RNA-levels of *Arhgef7*, the amount of F-actin, and deficits of synaptic transmission were normalized by Entinostat. However, repetitive behavior was not beneficially influenced [199].

Furthermore, increased levels of EHMT1 and EHMT2 were observed in mice of the models ex21|PRO (heterozygous) and ex4-9|ANK (homozygous), which lead to increased H3K9me2 di-methylation levels. This was then used as therapeutic target [156]. In the model ex21|PRO application of the highly potent and specific EHMT1/2 (histone methyltransferases) inhibitor UNC0642 induced a long-lasting improvement of social behavior, while di-methylation levels were also normalized. On a molecular level UNC0642 normalized the transcript-levels of 107 previously down-regulated and 84 previously up-regulated genes. Among those genes were *Homer1* and *Arc*. Additionally, H3K9me2-levels on the Arc-promotor were restored to WT-level. Interestingly, the RNA- and protein-level of *Grin1* and *Grin2a*, which had not been affected by SHANK3 deficiency, were upregulated upon UNC0642-treatment. Fitting this, the H3K9me2 occupancy at their respective promotor sites was reduced. On a physiological level UNC0642 also rescued NMDAR-mediated synaptic transmission. Notably, neither the beneficial effects of UNC0642 on social behavior, nor on synaptic transmission were present if an *Arc* shRNA-construct was co-administered, even inducing similar phenotypes in WT-mice. On the other hand, lentiviral knockdown of *Ehmt1* and *Ehmt2* in the PFC also normalized social behavior and synaptic transmission. UNC0642-treatment had no effect on repetitive behaviors. A general health screening did not show any major organic or behavioral side effects of UNC0642. Similarly, UNC0642 also rescued social preference and di-methylation levels in mice of the model ex4-9|ANK. The low-potency EHMT1/2 inhibitor BIX01294 only elicited a transient amelioration of social deficits in heterozygous ex21|PRO mice.

##### ARC-activation

Fitting some previously made assumptions derived from the observation of epigenetic dysregulation in SHANK3-deficient animals, local lentiviral activation of ARC in the PFC resulted in an increased social preference in heterozygous ex21|PRO mice [156].

##### Cofilin-inhibition

It has been described that heterozygous SHANK3 deficiency in ex21|PRO mice resulted in a reduction of cortical F-actin and an associated impairment of RAC1/PAK activity that led to an increased actin-depolymerization through increased activity of CFL1. Accordingly, inhibition of CFL1 via a brain-permeable CFL1 inhibitory peptide (TAT-p-cofilin peptide) was chosen as therapeutic strategy to rescue the phenotypes observed in these mice. The social deficits, repetitive behaviors, and NMDAR-hypofunction in the PFC and hippocampus were indeed alleviated by inhibiting CFL1 [177]. Notably, local injection into the PFC was sufficient to increase social preference and reduce self-grooming. These findings are in line with the previously made assumptions, concerning the affected pathways [177], and also findings from other studies [154].

##### RAC1-activation

In the same study that targeted CFL1 as downstream effector in the RAC1/PAK-pathway, a constitutively active form of RAC1 (CA-RAC1, HSV-delivered) was injected into the PFC of mutant ex21|PRO mice to achieve similar effects. Indeed, social deficits and NMDAR-hypofunction in pyramidal neurons of the PFC were also alleviated by activating RAC1. Notably, the observed deficits were inducible by inhibiting PAK or RAC1 in wild-type mice [177]. Thus, targeting actin regulators and specifically the RAC1/PAK/LIMK1/CFL1-pathway, which controls actin depolymerization, might provide a general strategy for future treatment of PMDS or SHANK3-associated ASD. Most interestingly, the RAC1-dependent and ARP2/3-mediated actin polymerization process [204] and the associated subunits or regulators of the WAVE regulatory complex, such as WASF1 [94], NCKAP1 [202], ABI1 [94] or BAIAP2 [202], were also dysregulated in models of SHANK3 deficiency and might be potential therapeutic targets to influence neuronal actin dynamics.

##### CLK2-inhibition

As phosphorylation of targets in the PI3K/AKT/MTOR-pathway was reduced in SHANK3-deficient ex21|PRO mice, CLK2, which induces the inactivation of AKT via activation of a PPP2CA regulatory subunit (PPP2R5B), was chosen as a possible therapeutic target. Indeed, CLK2 inhibition via application of TG003 was beneficiary in a SHANK3-deficient state, restoring sociability, reducing repetitive behaviors, and reinstating AKT-phosphorylation. However, TG003 had no influence on avoidance behavior [178].

##### PKA-inhibition

Since corticostriatal hyperactivity was observed in SHANK3-deficient mice of the model ex13-16|PDZ, PKIA as potent peptidergic inhibitor of the PKA catalytic subunit was expressed in medial striatal neurons to normalize their glutamatergic synapse maturation. Indeed glutamatergic neurotransmission was reduced by PKIA. On a behavioral level PKIA expression partly normalized repetitive behavior, anxiety-levels, and hypoactivity in these mice [183].

##### NTRK2-agonism

The NTRK2-agonist 7.8-DHF beneficially influenced spatial learning, but had no influence on social deficits, repetitive behavior, or hypoactivity in ex13-16|PDZ SHANK3-deficient mice [162].

##### Troponin-activation

Although muscular hypotonia is a pivotal and early-onset feature of the PMDS, treatments for this symptom have not been investigated frequently. A similar phenotype was observed in ex11|SH3 SHANK3-mutant mice, becoming manifest as an impaired neuromuscular performance in the hanging wire test, but also as morphological alterations of the spinal cord and the neuromuscular junction. The muscular dysfunction was partly normalized by Tirasemtiv (CK2017357), a fast-acting skeletal muscle troponin activator [111].

#### Genetic restoration

Because ASD is a neurodevelopmental disorder [3], it is of great interest whether some of the synaptic and behavioral impairments are reversible in adults. Anyhow it is probably beneficial to initiate treatment as early as possible.

Thus, the finding that adult restoration of SHANK3-expression in a cKI ex13-16|PDZ murine model reversed repetitive self-injurious grooming and social-interaction deficits is of great importance, although anxiety or motor coordination deficits were not alleviated. Furthermore, genetic restoration normalized deficitary neurotransmission, synaptic protein composition, and spine morphology. The behavioral deficits that were irreversible in adulthood were improved by early postnatal intervention and fully rescued by germline restoration [83]. Similarly, early genetic restoration in ex13|PDZ mice alleviated increased repetitive behaviors, social deficits, and hypoactivity [176]. Another study on Cre-mediated genetic rescue of *Shank3* during adulthood demonstrated an apparent amelioration of avoidance behavior, motor performance, and synaptic transmission in ex21|PRO-InsG3728 mice, while hypoactivity was not improved. However, the authors considered these results to be uninterpretable, since extensive control conditions revealed that some phenotypes were also “rescued” in Cre-positive but vehicle-treated mice, hinting at an effect of the Cre-transgene [187]. This study demonstrated that experiments, which are based on Cre-expression, should be carefully controlled.

Local genetic *Shank3*-restoration in the anterior cingulate cortex of adult ex13-16|PDZ cKI-mice was sufficient to normalize social deficits and synaptic protein levels, but only partly corrected alterations of synaptic transmission and spine density. Genetic restoration in this prefrontal area did not influence avoidance, repetitive, anxiety-like or hypoactive behavior [160]. Likewise, general, but also local restoration of *Shank3* in the medial PFC was sufficient to normalize social behavior and ameliorate encoding deficits concerning neuronal activity, which is induced by either self-experience or experiences made by conspecifics. The onset and progression of the normalization process of social behavior also correlated with the changes in neuronal encoding [196].

Most interestingly, specific cKI-mediated restoration of SHANK3 levels in cells of the caudal embryo or somatosensory neurons (ex13-16|PDZ-Cdx2^Cre^, -Advillin^Cre^) was sufficient to normalize somatosensory deficits and abnormal tactile sensorimotor gating, as well as social interaction or recognition, avoidance behavior, and anxiety-like behaviors. However, SHANK3-reexpression in these cells did not influence increased repetitive behaviors or cognitive deficits. Moreover, SHANK3-restoration in somatosensory neurons also normalized the number of PVALB-positive neurons in the somatosensory cortex and basolateral amygdala, but not in the visual cortex. Notably, Tamoxifen-induced Cre-expression in somatosensory neurons of ex13-16|PDZ-Advillin^CreERT2^ mice at P28 and thus SHANK3-restoration via cKI also led to normalized tactile sensorimotor gating and social interaction behavior. Reexpression of SHANK3 at this later timepoint did not influence certain abnormal somatosensory properties, social recognition, avoidance behavior, anxiety-like behaviors, and long-term recognition memory deficits. Late SHANK3-restoration in somatosensory neurons normalized the number of PVALB-positive neurons exclusively in the somatosensory cortex, but not in the basolateral amygdala and visual cortex, suggesting differing therapeutic windows to influence certain brain regions, which might also affect the extend of rescued phenotypes [140].

Considering the abovementioned findings, genetic restoration of SHANK3 levels or of downstream mediators during adulthood or earlier timepoints might relieve some of the synaptic and behavioral impairments, in a brain region and cell-specific mode.

#### Local neuronal activation

##### Prefrontal areas

As described above, genetic SHANK3 restoration specifically in the anterior cingulate cortex of adult ex13-16|PDZ cKI mice normalized social behavior and local synaptic protein levels, but only partly corrected alterations of synaptic transmission and spine density, and did not improve avoidance, repetitive, anxiety-like, or hypoactive behavior. Accordingly, optogenetic activation of this area normalized social behavior and anxiety-levels, but did not influence hypoactivity. Similar effects were observed upon DREADD-mediated activation of pyramidal cells localized in the anterior cingulate cortex, which rescued social deficits, but did not influence self grooming [160]. Another study reported that DREADD-mediated and *Camk2*-driven activation of the PFC normalized social behavior, NMDAR-mediated synaptic transmission, GRIN2A protein-levels, and *Sgk2* mRNA-levels. These beneficial effects were short-lasting and not present, if a TAT-peptide blocking SGKs was co-administered [197].

##### Dorsal striatum

In a study, which examined the differential effects of SHANK3 deficiency on either DRD1-positive MSNs of the direct/striatonigral pathway or DRD2-positive MSNs of the striatopallidal/indirect pathway in the dorsal striatum of ex13-16|PDZ mice, it was described that neurons of the indirect pathway were generally more affected. Fittingly, DREADD-mediated activation of these neurons normalized repetitive behaviors as measured by time spent self-grooming, while neither activation nor inhibition of DRD1-positive MSNs had significant effects on behavioral or motor phenotypes. A trend towards attenuated hypoactivity upon activation of MSNs of the striatonigral pathway was not statistically significant [182].

##### Dorsal raphe nucleus

Another study targeted the dorsal raphe nucleus as the major serotonergic nucleus to influence social interaction behavior in ex13-16|PDZ SHANK3-deficient mice. Optogenetic activation of these serotonergic neurons during a social interaction training paradigm enhanced social preference in SHANK3-deficient mice. The effect persisted for at least 10 days, if the training lasted for 3 consecutive days [165]. This finding might implicate that the application of certain therapeutics might benefit from an adjusted behavioral therapy that is carried out in parallel.

##### Ventral tegmental area

In the same study that investigated the serotonergic raphe nucleus, the VTA, representing a crucial part of the dopaminergic system, was also targeted via optogenetic activation. This did not influence the deficits of social behavior, but only induced a preference for certain spatial positions in the test cage [165].

#### Diet-based therapy

##### Zinc

One of the environmental factors, which are associated with an increased risk of ASD, is Zinc deficiency [53, 54]. As SHANK3 self-multimerizes in a Zinc-dependent manner via the SAM-domain [59, 102], Zinc supplementation has been investigated in the ex13-16|PDZ model of SHANK3 deficiency. Oral Zinc supplementation (150 ppm) for 6 weeks fully normalized phenotypes like social recognition, repetitive behavior, and anxiety-like behavior, but only partly rescued hypoactivity in these mice. Interestingly, Zinc supplemented mutant mice exhibited normalized NMDAR decay kinetics, while the amplitude of NMDAR-EPSCs, which had originally been intact, was decreased after Zinc supplementation. In line with this, functional striatal LTP induction was lost in mutant animals after supplementation, but not in WT mice. Additionally, an increased intensity of SHANK2 was observed in corticostriatal and thalamostriatal synapses [168]. Another study investigated chronic maternal Zinc supplementation (150 ppm) as an alternative strategy to prevent ASD-like phenotypes in ex13-16|PDZ SHANK3-mutant animals. Deficits of social behavior, increased repetitive behavior, and elevated anxiety levels were permanently reversed by maternal Zn supplementation. Striatal NMDAR-mediated synaptic transmission and presynaptic short-term plasticity were rescued as well, while the AMPAR-mediated transmission was still impaired. Notably, maternal Zn supplementation resulted in an impaired AMPAR- and NMDAR-mediated transmission in wild-type animals, without affecting their behavior [164].

##### Lactobacillus reuteri

Besides Zinc supplementation, another diet-based therapy studied in the murine model ex13-16|PDZ is the oral substitution of *Lactobacillus reuteri* specimen. Treatment with *L. reuteri* influenced social behavior beneficially [161, 211], and normalized induced synaptic transmission and plasticity in the VTA [161], while hypoactivity [161] or anxiety-like behavior [211] were not influenced. Notably, the beneficial effects of *L. reuteri* were dependent on vagal signals to the brain and OXTR-signaling [161].

## Conclusions and outlook

### Validity and role of animal models in PMDS research

Although different genetic modifications were applied in animal models of SHANK3 deficiency, especially exon-specific KO strategies (ex4-22|ALL, ex4-9|ANK, ex4-7|ANK, ex9|ANK, ex11|SH3, ex13|PDZ, ex13-16|PDZ, ex14-16|PDZ, ex21|PRO, ex11-21|SH3-PRO) result in similar phenotypes with varying severity. It had been hypothesized that the disruption of specific exons would result in diverging behavioral phenotypes, but such a clear isoform-related pattern cannot be observed in the literature reviewed. Regardless of which exons were targeted, all major ASD-related phenotypes have been reported in these SHANK3-deficient models across multiple studies.

However, the absence of such distinct patterns might not indicate the non-existence of such differential phenotypes in general, but rather imply that commonly applied testing paradigms lack specificity and sensitivity. Customizable and highly automatized analysis of freely interacting groups of mice enabled by solutions based on machine learning and other techniques, which should ideally be open-source, could reveal more nuanced, but also more comparable behavioral phenotypes in the future [155, 157, 159, 234, 235]. On the other hand, this lack of clear isoform-specific patterns could hint at the properties of ASD pathophysiology in general. Unlike a defined disorder, which follows the classical order of an underlying etiology and subsequently resulting clinical abnormality, ASD might rather be a compensatory process that emerges as the common outcome of various disturbances impacting neuronal physiology. The question whether ASD should be regarded as a compensatory mechanism to tackle multiple abnormalities of the nervous system is also raised by the apparent functional heterogeneity of ASD risk genes [14, 15]. If the appearance of ASD would rely at least partly on the initiation of compensatory mechanisms, the molecular changes, which can be observed in animal models of SHANK3 deficiency or other models of ASD, should not be principally regarded as disruptive, because such changes might be part of beneficial mechanisms. These could also represent potential therapeutic targets. Nevertheless, some studies suggest that the SHANK3-isoform composition contributes to the severity and reproducibility of the observed deficits. For instance, the model ex13-16|PDZ, which lacks the hypothetical protein isoforms SHANK3a-d, showed a more robust phenotype than ex4-9|ANK mice [155], lacking SHANK3a-b. Direct comparisons of SHANK3-deficient models are needed to eliminate confounding factors like methodological variability, which limit the comparability in the current literature. Such studies might then be able to elucidate isoform-specific functions, but also the compensatory mechanisms between them. Generally, full deletion models of SHANK3 encompassing exons 4-22 (ex4-22|ALL) and thus lacking all isoforms facilitate the study of SHANK3’s basic biological functions by avoiding inter-isoform compensation. Such models also provide an improved construct validity regarding PMDS with a full deletion of *SHANK3*.

Additionally, the investigation of point/missense mutations provides an interesting option to study modular functions of SHANK3, as these animal models were shown to only recapitulate specific phenotypes. For instance, concerning the major ASD-related phenotypes the model ex8|ANK-Q321R only showed increased repetitive behavior [132], while in the model ex17|PRM-S685I, affecting the ABI1-binding site, only social behavior was deficient [94]. An ASD-related mutation introduced in animals of the model ex21|PRO-InsG3680 had more general effects, resulting in abnormal social behavior, increased stereotyped behaviors, elevated anxiety-levels, and impaired motor and sensory function [127]. Similar behavioral domains were affected in mice harboring a schizophrenia-associated mutation (ex21|PRO-R1117X), but these animals did not show stereotypies [127]. Notably, ex21|PRO-InsG3680 and -R1117X result in different isoform patterns with an almost complete deletion of SHANK3, and the expression of a truncated protein, respectively, which might be causative for their differing phenotype concerning stereotyped behaviors and subphenotypes of social behavior [127].

Conditional and thus cell-type-specific deletion of SHANK3 has also been insightful, concerning the cellular and regional foundation of certain deficits observed in constitutive deletion models. Targeted SHANK3 deletion in somatosensory neurons or the caudal part of the embryo and neural tube (ex13-16|PDZ-Advillin^Cre^ or -Cdx2^Cre^ [140]) was sufficient to induce social deficits, avoidance behavior, anxiety, and sensory dysfunction in these mice, but did not result in stereotyped behavior or cognitive deficits. When either inhibitory (ex14-16|PDZ-Viaat^Cre^ [131], ex4-22|ALL-Dlx5/6^Cre^ [129]) or excitatory neurons (ex14-16|PDZ-Emx1^Cre^ [133], ex4-22|ALL-NEX^Cre^ [129]) were targeted seperately, stereotyped behavior was consistently observed in all models, while elevated anxiety levels were only present if GABAergic neurons were targeted. Finally, differential deletion of SHANK3 either affecting DRD1- or DRD2-positive neurons (ex4-22|ALL-Drd1^Cre^ or -Drd2^Cre^ [129]) resulted in a minor anxiety-like phenotype and increased repetitive behavior, respectively. Neither of them resulted in deficient social behavior. Importantly, one study [140] also investigated the impact of different timepoints of inducing postnatal SHANK3 deficiency in the model ex13-16|PDZ-Advillin^CreERT2^. Induction on postnatal day 5 (P5) recapitulated all major phenotypes observed in animals with prenatal Cre-expression, including abnormal social interaction and recognition, avoidance behavior, increased anxiety-levels, impaired sensorimotor gating and somatosensory function, and reduced numbers of PVALB-positive neurons in the somatosensory cortex and the amygdala. When induced at P10, SHANK3 deficiency did not result in avoidance behavior, anxiety, or an abnormal number of PVALB-neurons in the amygdala. Lastly, tamoxifen-mediated induction at P28 only resulted in abnormal social recognition, impaired sensorimotor gating and somatosensory function, and reduced numbers of PVALB-positive neurons in the somatosensory cortex. These results underline the importance of the developmental aspect of SHANK3-dependent phenotypes.

In summary, many deficits that were observed in SHANK3-deficient animals recapitulate symptoms that are observed in PMDS patients, ranging from ASD-related phenotypes such as social deficits or repetitive behaviors, to motor and sensory abnormalities resembling hypotonia or impaired nociception in human patients. Thus, such models provide a valid and valuable instrument to advance our understanding of the neurobiological basis of PMDS as SHANK3-associated syndrome, but also ASD or the functioning of the CNS and other organs in general.

### Translational implications for PMDS treatment

A wide array of therapeutic strategies has been applied in animal studies investigating SHANK3 deficiency in rodents, ranging from mechanisms targeting glutamatergic neurotransmission to epigenetic regulation of gene expression. Although some of these treatments showed promising results, it has to be noted that a comprehensive screening for side effects has only rarely been reported. Considering the fact that the different compounds or strategies, and also timepoints of intervention had differential effects on the observed abnormalities, it seems likely that a combination of therapies might be beneficial. However, most of the pharmaceutical compounds or other strategies described in the sections above have not translated into clinical routine, although there has been preliminary evidence of beneficial effects for some of them in PMDS-patients. Thus, further clinical studies have to be conducted to strengthen the evidence. Several patient studies are underway to establish therapies, which might alleviate core symptoms of PMDS and increase quality of life in general. Despite the fact that the PMDS affects multiple organs, including the musculoskeletal or gastrointestinal system, most of these studies focus on neuropsychiatric symptoms. Therapies, which would improve symptoms associated to organs other than the CNS, should also be investigated, as they often affect everyday quality of life.

#### Treatments used in clinical practice

Generally, there is no specific treatment option for PMDS-patients. Clinical management of these patients focuses on comorbidities like epilepsy, for which general treatment guidelines are already available. The neuropsychiatric domain of PMDS, which also includes ASD, is approached according to the presently recommended strategies. These include parent-mediated, school-based, and therapist-delivered interventions. A primary objective should also be to support independence of the patients during adulthood. Medication can ameliorate symptoms caused by comorbidities like epilepsy, irritability, hyperactivity, and anxiety, but pharmacological therapy of core symptoms is difficult and an active field of research [3, 236–238].

Compounds, which have been shown to relieve some of the symptoms observed in PMDS patients specifically, include IGF1, insulin, lithium, and risperidone. Early studies reported beneficial effects of IGF1 on the synaptic transmission of patient-derived neurons [239] and on ASD-associated behaviors in PMDS patients [240]. The effects of IGF1 are now further investigated in a phase 2 trial (NCT01525901). Subtle effects were described for insulin, which improved motor development, cognition, and social skills [241, 242]. In a case report, low-dose risperidone alleviated psychomotor agitation, aggression, anxiety, and insomnia, which had previously been described in this PMDS-patient [243]. More recently, several case reports on the effects of lithium in PMDS-patients with a comorbid catatonia or bipolar disorder demonstrated a stabilization of mood and affect, reversal of the catatonia features, and an improvement of regression [244–246]. The efficacy of lithium is currently further studied in a phase 3 trial (NCT04623398). Other substances like recombinant human growth hormone (NCT04003207, completed phase 2 trial), OXT (NCT02710084, completed phase 2 trial, undergoing peer-review), Q10 Ubiquinol (NCT04312152), and the inhibitor of the RAS/RAF/MAPK pathway AMO-01 (NCT03493607, PMDS with epilepsy) are currently investigated or undergo peer-review for publication.

#### Future perspectives

Genetic restoration of *Shank3* in rodents has been shown to reverse core deficits even in adult animals [83, 140, 160, 176]. Direct gene targeting in humans seems plausible as future treatment option for certain forms of ASD as the recent success of many techniques associated to the detection and treatment of genetic disorders might provide the neccessary tools. Especially therapies developed for spinal muscular atrophy type 1 [247, 248], or the manipulation of gene products rather than the DNA itself [249, 250] like targeting haploinsufficiencies by CRISPR-mediated gene activation via promotors or enhancers [249], might serve as a proof of principle for other genetic disorders. Early-onset therapy, which might be crucial concerning ASD, will also be facilitated by the detection of mutations in utero and during early postnatal development, which is now possible [3]. Defects with a large effect seen in syndromes such as the PMDS or fragile X syndrome, but also mutations affecting high confidence ASD risk genes such as *SCN2A* and *CHD8* [14, 15] will represent primary targets of such new therapies [3]. With regard to *SHANK3*-insufficiency it has to be noted that the resulting behavioral phenotype is highly sensitive to gene dosage, which has been demonstrated in the abovementioned studies on SHANK3 deficiency or overexpression. Thus, the increase or decrease of expression induced by genetic targeting of *SHANK3* in humans would have to be rather precise to enable individual dose adjustments to avoid side effects reminiscent of either manic- or ASD-like behavior, respectively.

Additionally, compounds that showed promising results in preclinical animal studies, such as epigenetic targeting via histone deacetylase inhibitors [154, 156, 198, 199] or modulation of peripheral GABAAR [140], should be tested in the setting of clinical trials to evaluate their efficacy in humans. In the long-term, the combination of multiple treatment strategies seems to be necessary, as none of the proposed compounds influences all domains affected in human patients.

New approaches in preclinical animal studies should take into account the evidence for certain therapeutic windows to influence specific circuits and the associated behavioral phenotype [140], but also that it might be possible to reopen those critical periods of plasticity to enhance treatment efficacy. For instance, it has been reported recently, that the reopening of a critical period for social reward learning, which depends on OXT-mediated LTD in the nucleus accumbens, can be established by 3,4-methylenedioxymethamphetamine (MDMA) [251]. Thus, behavioral abnormalities, which are otherwise largely insusceptible to pharmaceutical compounds or behavioral interventions due to certain developmental time frames limiting neuronal plasticity, might be influencable by utilizing a sequential combination of therapeutic strategies including MDMA. In general, the goal of any translational and clinical scientific approach to symptoms of SHANK3 deficiency and the PMDS should be to address the complaints expressed by patients or their parents. This does not necessarily imply the normalization of their behavioral traits. Also apart from neuroscientific questions addressing ASD, more research is needed on other symptoms like sleep disturbances and gastrointestinal problems, which frequently occur in these patients.

## Appendix

For the programming of the circular barplots a database containing abnormality scores (1 = no change, 2 = abnormal with respect to control condition) for all the experiments, which were reported in the literature on SHANK3-deficient animals included in this review (total of 94 publications, summarized in Table 2), was generated. Every data point was assigned to a category (Structure & Function, Behavior, Neurology), a subcategory (Structure & Function: Molecular, Physiology, Morphology; Behavior: Social, Stereotypies, Other; Neurology: Motor, Sensory, Cognition, Other), and an interpretation (e.g., protein composition, repetitive behavior, or somatosensory function; also see Table 1 for the full list). To compile the plots, the data was pooled according to the exons targeted and homo- or heterozygosity. Finally the mean was calculated for each interpretation and displayed as circular barplots. Notably, data was summarized in such a way, even if the same exons were targeted in independently published strains (e.g., ex4-22|ALL [128, 130]). As studies on macaques either investigated individuals or genetically heterogenous groups, the data was pooled according to species (*Macaca fascicularis*). No statistical tests were applied, since this approach was used for visualization purposes only. R [253] was used to generate plots. Additional packages used were tidyr [254], plyr [255], dplyr [256], reshape2 [257], and ggplot2 [258].

**Table 2:** Summary of the models and respective literature used to generate the database. Experiments from a total of 94 publications on SHANK3 deficiency were included in the database and categorized as described in Table 1

Code	Strategy	Addition	Literature
ex4-22|ALL	KO	Murine	HET [92, 128, 130]; HOM [92, 128–130]
ex4-9|ANK	KO	Murine	HET [78, 80, 122, 124, 167, 190, 200, 207, 209, 218, 220]; HOM [78, 80, 124, 128, 140, 154–156, 167, 177, 190, 209, 218, 220, 252]
ex4-7|ANK	KO	Murine	HOM [90]
ex9|ANK	KO	Murine	HOM [82, 175]
ex11|SH3	KO	Murine	HET [123, 219]; HOM [111, 118, 123, 157, 193, 202, 203, 212, 219]
ex13|PDZ	KO	Murine	HET [139, 176]; HOM [139, 176]
ex13-16|PDZ	KO	Murine	HET [106, 140, 151, 166, 181, 188, 201, 209, 213, 214]; HOM [90, 94, 140, 151, 155, 158–166, 168, 181–183, 188, 189, 192, 205, 206, 208, 209, 211, 213–215]
ex13-16|PDZ	cKI	Murine; Genetic restorationvia Cre-recombinase	HET [140, 196]; HOM [83, 160]
ex14-16|PDZ	KO	Murine	HOM [131, 133]
ex21|PRO	KO	Murine	HET [154, 156, 177–179, 194, 197–199]; HOM [125, 177–179, 191]
SHANK1 +SHANK3-ex11|SH3	KO	Murine	HOM [138]
ex6|ANK	KO	Rat	HET [134, 180, 216, 217]; HOM [134, 180, 217]
ex11-21|SH3-PRO	KO	Rat	HET [135]; HOM [135]
ex6,ex12|ANK,SH3	KO	Macaque	HET [137, 227]
ex21|PRO	KO	Macaque	HET or HOM [136]
ex8|ANK-Q321R	KI	Murine; Missense point mutation	HET [132]; HOM [132]
ex17|PRM-S685I	KI	Murine; Missense point mutation	HET [94]; HOM [94]
ex21|PRO-InsG3680	KI	Murine; Frameshift mutation	HET [127]; HOM [127, 210]
ex21|PRO-R1117X	KI	Murine; Non-sense point mutation	HET [127]; HOM [127]
ex21|PRO-InsG3728	KI	Murine; Frameshift mutation	HET [126, 187]; HOM [126, 187]
ex4-22|ALL-NEX^Cre^	cKO	Murine; Affecting neocortical excitatory neurons	HOM [129]
ex4-22|ALL-Dlx5/6^Cre^	cKO	Murine; Affecting GABAergic forebrain neurons	HOM [129]
ex4-22|ALL-Drd1^Cre^	cKO	Murine; Affecting DRD1-positive neurons	HOM [129]
ex4-22|ALL-Drd2^Cre^	cKO	Murine; Affecting DRD2-positive neurons	HOM [129]
ex4-22|ALL-Nav1.8^Cre^	cKO	Murine; Affecting Nav1.8-positive cells	HET [92]; HOM [92]
ex13-16|PDZ-Advillin^Cre^	cKO	Murine; Affecting somatosensory neurons	HET [140]; HOM [140]
ex13-16|PDZ-Cdx2^Cre^	cKO	Murine; Affecting caudal embryonic lineage	HET [140]
ex14-16|PDZ-Viaat^Cre^	cKO	Murine; Affecting GABAergic cells	HOM [131]
ex14-16|PDZ-Emx1^Cre^	cKO	Murine; Affecting excitatory neurons and glia	HOM [133]
SHANK3-eGFP	TG	Murine; Overexpression of SHANK3	HEM [93, 96, 151, 205, 228–231]

*KO* knockout, *KI* knockin, *cKO* conditional KO, *cKI* conditional KI, *TG* transgenic, *HET* heterozygous, *HOM* homozygous, *HEM* hemizygous

Abbreviations and the writing format for genes and proteins were chosen according to the guidelines of the HUGO Gene Nomenclature Committee for humans and the Mouse Genome Informatics for rodents. They are not included in the abbreviations list, as they are regarded as symbols.

## Data Availability

The database, which was generated for the programming of the circular barplots, is available upon request.

## Abbreviations

ASD: Autism spectrum disorder
SHANK: SH3 and multiple ankyrin repeat domains
ProSAP: Proline-rich synapse-associated protein
PMDS: Phelan-McDermid syndrome
DSM-5: Fifth edition of the American Psychiatric Association’s Diagnostic and Statistical Manual of Mental Disorders
ADHD: Attention-deficit hyperactivity disorder
CNV: Copy number variation
PSD: Postsynaptic density
CNS: Central nervous system
PNS: Peripheral nervous system
PDZ: PSD95/DlgA/Zo-1
SH3: Src homology 3
SPN: SHANK/ProSAP/N-terminal
PRO: Proline-rich
SAM: Sterile alpha motif
ANK: Ankyrin repeats
MSN: Medium spiny neuron
PRM: Proline-rich motif
PFC: Prefrontal cortex
VTA: Ventral tegmental area
PAM: Positive allosteric modulator
OXT: Oxytocin
MDMA: 3,4-Methylenedioxymethamphetamine
